# Supplementary material for: Using machine learning to identify important predictors of COVID-19 infection prevention behaviors during the early phase of the pandemic
Source: Patterns (N Y). 2022 Mar 9;3(4):100482. doi: 10.1016/j.patter.2022.100482 (PMC8904175; doi:10.1016/j.patter.2022.100482)

Patterns, Volume *3*

Supplemental information

Using machine learning to identify important predictors of COVID-19 infection prevention behaviors during the early phase of the pandemic

Caspar J. Van Lissa, Wolfgang Stroebe, Michelle R. vanDellen, N. Pontus Leander, Maximilian Agostini, Tim Draws, Andrii Grygoryshyn, Ben Gützgow, Jannis Kreienkamp, Clara S. Vetter, Georgios Abakoumkin, Jamilah Hanum Abdul Khaiyom, Vjolica Ahmedi, Handan Akkas, Carlos A. Almenara, Mohsin Atta, Sabahat Cigdem Bagci, Sima Basel, Edona Berisha Kida, Allan B.I. Bernardo, Nicholas R. Buttrick, Phatthanakit Chobthamkit, Hoon-Seok Choi, Mioara Cristea, Sára Csaba, Kaja Damnjanović, Ivan Danyliuk, Arobindu Dash, Daniela Di Santo, Karen M. Douglas, Violeta Enea, Daiane Gracieli Faller, Gavan J. Fitzsimons, Alexandra Gheorghiu, Ángel Gómez, Ali Hamaidia, Qing Han, Mai Helmy, Joevarian Hudiyana, Bertus F. Jeronimus, Ding-Yu Jiang, Veljko Jovanović, Željka Kamenov, Anna Kende, Shian-Ling Keng, Tra Thi Thanh Kieu, Yasin Koc, Kamila Kovyazina, Inna Kozytska, Joshua Krause, Arie W. Kruglanksi, Anton Kurapov, Maja Kutlaca, Nóra Anna Lantos, Edward P. Lemay Jr., Cokorda Bagus Jaya Lesmana, Winnifred R. Louis, Adrian Lueders, Najma Iqbal Malik, Anton P. Martinez, Kira O. McCabe, Jasmina Mehulić, Mirra Noor Milla, Idris Mohammed, Erica Molinario, Manuel Moyano, Hayat Muhammad, Silvana Mula, Hamdi Muluk, Solomiia Myroniuk, Reza Najafi, Claudia F. Nisa, Boglárka Nyúl, Paul A. O'Keefe, Jose Javier Olivas Osuna, Evgeny N. Osin, Joonha Park, Gennaro Pica, Antonio Pierro, Jonas H. Rees, Anne Margit Reitsema, Elena Resta, Marika Rullo, Michelle K. Ryan, Adil Samekin, Pekka Santtila, Edyta M. Sasin, Birga M. Schumpe, Heyla A. Selim, Michael Vicente Stanton, Samiah Sultana, Robbie M. Sutton, Eleftheria Tseliou, Akira Utsugi, Jolien Anne van Breen, Kees Van Veen, Alexandra Vázquez, Robin Wollast, Victoria Wai-Lan Yeung, Somayeh Zand, Iris Lav Žeželj, Bang Zheng, Andreas Zick, Claudia Zúñiga, and Jocelyn J. Bélanger

Note. Some tables are too large to fit into a document and are thus linked separately. For the machine learning analysis, all analysis code and results are provided online at: DOI: 10.5281/zenodo.5948816. The PsyCorona survey details, including translation procedures and codebook in 30 languages, is available on the Open Science Framework at https://osf.io/qhyue/

Translation Procedures

Table S1. *PsyCorona Scale Translation Procedure*

Language	Translators	Backward Translation	Translation & Revision Team	Translation software	Other Translation Methods
Albanian	2	Yes	Yes	Yes	Backward translation used for some items only to check meaning.
Arabic	2	No	Yes	Yes	
Bengali	1	No	No	Yes	
Croatian	2	No	Yes		
Dutch	2	No	Yes	Yes	
English	*N/A*	*N/A*	*N/A*		*Survey and scales were developed in English*
Farsi	3	No	Yes	Yes	Backward translation used for some items only to check meaning, Initial survey with 3 translators, follow-up survey by 1 translator
French	2	No	Yes	Yes	
German	3	No	Yes	Yes	Searched for scales in papers and databases
Greek	2	No	Yes		
Hindi	1	No	No	Yes	
Hungarian	4	No	Yes		
Indonesian	2	No	Yes		Assisted using online dictionaries
Italian	2	No	Yes	Yes	
Japanese	2	No	Yes		
Korean	2	No	Yes		
Malay	2	No	Yes		Assisted using online dictionaries
Polish	4	Yes	Yes	Yes	
Portuguese	1	No	No	Yes	
Romanian	2	Yes	Yes		

Russian	2	No	Yes		
Serbian	2	No	Yes		
Simplified Chinese	2	No	Yes		
Spanish	4	Yes	Yes		
Thai	2	Yes	Yes		
Traditional Chinese	2	No	Yes		
Turkish	3	No	Yes		
Ukrainian	2	No	Yes	Yes	Backward translation used for some items only to check meaning.
Urdu	2	Yes			
Vietnamese	2	Yes	Yes	Yes	Assisted using online dictionaries

Note: Translators = Number of translators who worked on this scale. Backward Translation = One person translated the measure from English to the language, and a different person translated the scale from the language back to English to check for scale meaning. Translation & Revision Team = One person translated the scale from English to the language, and a second person revised this translation. Alternatively, each person translated the scale and worked together during the revision. Translation Software = Translators used a translation software in the process (e.g., Google Translate). Other Translation Methods = other methods used in the translation of the survey.

Table S2. *Potentially relevant translation issues*

Language	Translation Issues
Albanian	• We were careful to choose semantically correct translations over more literal ones aiming to accommodate cultural differences. In some cases it was needed to add more words for correct understanding.<br>• "Online vs. offline contact" was translated as "online vs. direct contact"<br>• The item about “belief in one God/more than one God” was translated as “belief in one God” as all the religions in Kosovo and Albania are monotheistic. While these kinds of beliefs were not separated for ex. in two items but were within one item, it may have been confusing for the subjects so the translation was adapted culturally.<br>• Items pertaining to political orientation (left/right wing) may not be relatable due to the terminology used. Longer descriptions may have been needed to explain the terms and ensure they are correctly understood.
Arabic	• Some words/phrases were changed or removed to accommodate regional religious beliefs.<br>• There wasn’t a word for ‘local community’ in Arabic so used the term ‘society’ instead.<br>• In the question where there is a distinction between should and do isolate/social distance myself, and want/have to … a formatting error caused the wrong term to be bolded in some items, but the wording was the same.
Bengali	*None*
Croatian	• Difficulty translating formidability items as the word formidability does not translate well. We adjusted the translation for better understanding.<br>• QID536 - “The events in my life are mainly determined by own actions” - we translated this as “The events in my life are mainly under my control” as this is more semantically correct.
Dutch	• Formidability was translated as 'powerful' as the Dutch word for formidability is rarely used.<br>• "Online vs. offline contact" was translated as "online vs. face-to-face contact"
English	*N/A*
Farsi	• Multiple questions did not translate well.<br>• Attitude about politics is a relatively western way to categorize people into groups.
French	*None*
German	• Some items were hard to translate. E.g. ‘community’ does not translate well.<br>• Semantically correct translations were sometimes chosen over more literal ones to accommodate cultural differences.
Greek	• “Online” in the item “In the past 7 days, how many days did you have **online** (video or voice) contact with …” was translated “Internet”.<br>• “Community” (in the present context) does not translate well into Greek.
Hindi	• Some items were too technical and did not translate well, so simpler translations conveying the meaning were chosen.
Hungarian	• Some items were difficult to translate accurately.
Indonesian	• Some items were difficult to translate accurately due to the inequality of meanings.

Italian	*None*	
Japanese	*None*	
Korean	*None*	
Malay	•	Some items were difficult to translate literally due to cultural considerations. E.g., the item about belief in one God/more than one God may be perceived as offensive to Malay Muslim when the item is being written as one item. Agreeing on the item may indicate that the individual believes in either one and this is unacceptable to the majority of Muslims in Malaysia.<br>• Items pertaining to political orientation (left/right wing) may not be relatable to many locally, due to the terminology used. Longer descriptions may be needed to explain the term to ensure the terms could be understood correctly.
Polish	•	Tightness-looseness construct was difficult to translate.
Portuguese	*None*	
Romanian	*None*	
Russian	•	Tightness-looseness construct is difficult to express in Russian.<br>• The terms "economic left-right" and "libertarian-authoritarian" make little sense without explanation to most Russians.
Serbian	•	Difficulty translating formidability items.<br>• Identification item translated as *I feel close to* instead of *I identify with*.
Simplified Chinese	*None*	
Spanish	•	Care taken when finding equivalence between standard Spanish and Latin American Spanish.
Thai	•	Difficulty in translating cross-cultural research terms.<br>• Some items were difficult to translate literally and accurately.<br>• Questions about bodies were confusing.
Traditional Chinese	•	Translated “in my country” to “in the place I live” in order to accommodate both Taiwan and Hong Kong (which is not a country, but a special administrative region).
Turkish	*None*	
Ukrainian	•	Questions about ‘bodies’ were confusing since the metaphor itself might not have been fully clear for the local population.<br>• The same concerns formidability. Many sentences had to be restructured in order to save the meaning of the question.
Urdu	*None*	
Vietnamese	•	Some translated items were difficult to express accurately in Vietnamese due to political and social issues (eg. protest/ protesting) and some were not popular to most Vietnamese people (eg. economic left-right or libertarian - authoritarian).

Table S4. Samples in the 28 countries that remained in the data after cleaning.

Country	n	Female	Male	Gender: other	Primary education	Secondary education	Vocational education	Some higher edu	Bachelor's degree	Master's degree	PhD
Ukraine	1433	0.603	0.396	0.001	0.004	0.091	0.134	0.386	0.108	0.22	0.057
Italy	2006	0.602	0.393	0.004	0.006	0.064	0.052	0.503	0.116	0.216	0.043
Greece	2875	0.675	0.323	0.002	0.006	0.017	0.048	0.25	0.379	0.232	0.068
Romania	2704	0.609	0.388	0.003	0.013	0.242	0.032	0.252	0.283	0.154	0.024
Indonesia	2410	0.509	0.486	0.005	0.009	0.351	0.06	0.048	0.37	0.128	0.034
Malaysia	895	0.712	0.286	0.002	0.002	0.056	0.009	0.12	0.534	0.229	0.049
Philippines	1530	0.564	0.425	0.011	0.01	0.077	0.065	0.108	0.555	0.126	0.058
Argentina	1412	0.565	0.431	0.004	0.01	0.233	0.142	0.28	0.241	0.053	0.041
Russia	1438	0.612	0.384	0.003	0.004	0.079	0.195	0.45	0.088	0.133	0.05
USA	11048	0.62	0.373	0.007	0.033	0.094	0.056	0.196	0.389	0.179	0.053
Canada	1538	0.574	0.416	0.01	0.02	0.174	0.109	0.205	0.31	0.141	0.042
Japan	1326	0.474	0.522	0.004	0.002	0.173	0.039	0.334	0.371	0.059	0.02
Egypt	1158	0.841	0.157	0.002	0.007	0.196	0.026	0.477	0.246	0.034	0.012
Netherlands	2409	0.623	0.371	0.007	0.018	0.122	0.183	0.222	0.133	0.234	0.088
Saudi Arabia	1468	0.527	0.463	0.01	0.015	0.192	0.061	0.101	0.493	0.099	0.039
France	1801	0.581	0.414	0.005	0.027	0.145	0.195	0.186	0.111	0.189	0.147
Spain	3203	0.627	0.368	0.006	0.014	0.12	0.158	0.299	0.253	0.106	0.05
Germany	1690	0.565	0.43	0.005	0.011	0.109	0.314	0.179	0.133	0.202	0.053
United Kingd	1935	0.612	0.383	0.005	0.008	0.193	0.132	0.191	0.258	0.158	0.06
South Korea	1452	0.57	0.427	0.003	0.005	0.03	0.015	0.403	0.421	0.097	0.03
Turkey	1826	0.604	0.395	0.002	0.008	0.015	0.208	0.107	0.465	0.153	0.045
Kazakhstan	812	0.562	0.437	0.001	0.001	0.041	0.041	0.302	0.268	0.268	0.079
Australia	1216	0.535	0.46	0.005	0.013	0.22	0.164	0.171	0.296	0.101	0.034
Kosovo	830	0.838	0.162	0	0.004	0.078	0.045	0.299	0.345	0.195	0.034
Brazil	1395	0.577	0.422	0.001	0.02	0.241	0.092	0.339	0.182	0.096	0.029
Poland	718	0.832	0.154	0.014	0.014	0.331	0.059	0.089	0.12	0.331	0.055
Republic of S	2122	0.661	0.337	0.002	0.013	0.17	0.268	0.121	0.248	0.141	0.039
South Africa	1422	0.568	0.429	0.004	0.017	0.189	0.071	0.361	0.283	0.061	0.018

Table S5: Scale descriptive statistics after combining PsyCorona survey items

Subscale	Items	n	mean	sd	min	max	skew	skew_2s	kurt	kurt_2se	Reliabili	Interpret	min_load	max_load
disc	3	55979	0.64	0.77	-2	2	-0.45	-21.51	0.15	3.55	0.68	Questionable	0.41	0.84
jbinsec	4	46018	-0.42	1.08	-2	2	0.38	16.71	-0.57	-12.39	0.81	Good	0.63	0.88
pfs	3	55962	0.12	1.05	-2	2	-0.1	-4.77	-0.66	-15.95	0.85	Good	0.65	0.92
fail	3	55981	-0.01	0.86	-2	2	-0.02	-0.92	-0.18	-4.37	0.66	Questionable	0.48	0.72
lone	3	56005	2.39	1.02	1	5	0.43	20.68	-0.51	-12.33	0.82	Good	0.76	0.82
probsolving	3	55976	3.7	0.85	1	5	-0.48	-23.35	0.15	3.63	0.84	Good	0.77	0.86
posrefocus	3	55973	3.1	0.91	1	5	-0.16	-7.88	-0.17	-4.1	0.85	Good	0.75	0.85
c19proso	4	55979	0.84	1.23	-3	3	-0.55	-26.37	0.16	3.84	0.77	Acceptable	0.56	0.8
c19perbeh	3	55982	2.19	1	-3	3	-1.88	-91.01	4.45	107.54	0.75	Acceptable	0.59	0.95
c19rca	3	55975	1.48	1.34	-3	3	-1.01	-48.75	0.78	18.81	0.71	Acceptable	0.59	0.81
ecoproso	4	55910	0.63	1.33	-3	3	-0.51	-24.82	0.05	1.13	0.86	Good	0.67	0.84
ecorca	3	55900	-0.1	1.41	-3	3	-0.15	-7.13	-0.43	-10.36	0.65	Questionable	0.59	0.68
bordeom	3	55970	1.37	1.29	-3	3	0	0.19	-0.35	-8.53	0.53	Poor	0.07	0.96
migrantthreat	5	55643	5.43	2.32	1	10	-0.18	-8.82	-0.68	-16.25	0.91	Excellent	0.75	0.89
cognitive test	3	55963	1.96	0.38	1	3	-0.78	-37.56	-0.3	-7.2	0.27	Unacceptable	0.22	0.47
neuro	3	55946	0.08	1.3	-3	3	-0.02	-1.21	-0.38	-9.11	0.69	Questionable	0.55	0.86
para	3	55901	3.93	2.19	0	10	0.29	14.02	-0.21	-4.96	0.69	Questionable	0.41	0.91
consp	3	55710	6.56	2.1	0	10	-0.46	-22.25	-0.03	-0.69	0.73	Acceptable	0.47	0.83

Fig. S1. Distribution of participation dates.

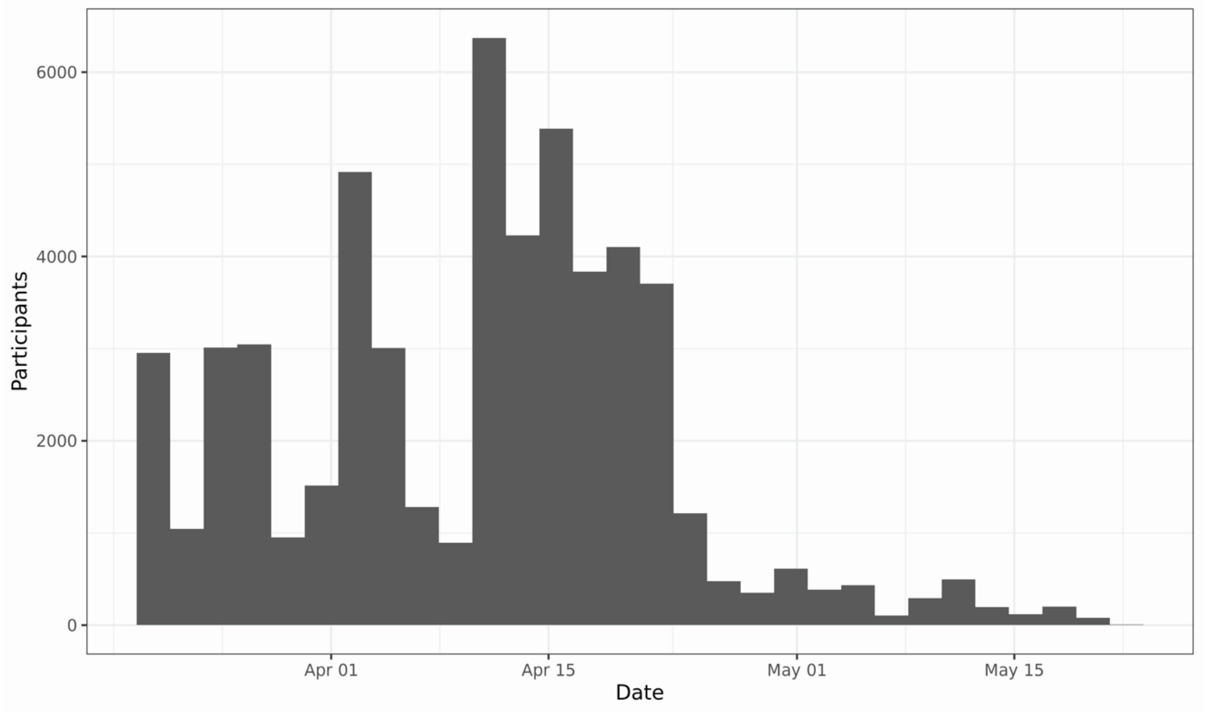

Supplement: Document S1. Figure S1 and Tables S1, S2, S4, and S5 [file mmc1.pdf]
