# Supplementary material for: Using machine learning to identify important predictors of COVID-19 infection prevention behaviors during the early phase of the pandemic
Source: Patterns (N Y). 2022 Mar 9;3(4):100482. doi: 10.1016/j.patter.2022.100482 (PMC8904175; doi:10.1016/j.patter.2022.100482)

Article

Patterns

Using machine learning to identify important predictors of COVID-19 infection prevention behaviors during the early phase of the pandemic

Highlights

- We studied predictors of COVID-19 prevention behaviors in a cross-national study
- The strongest predictors related to injunctive norms

Authors

Caspar J. Van Lissa, Wolfgang Stroebe, Michelle R. vanDellen, ..., Andreas Zick, Claudia Zúñiga, Jocelyn J. Bélanger

Correspondence

c.j.vanlissa@uu.nl

In brief

In a study of 56,072 participants from 28 countries, we used a machine-learning approach to identify the strongest predictors of COVID-19-infection-prevention behavior (pre-vaccine). Few country-level data variables predicted outcomes. Instead, individual psychological variables predicted outcomes. Injunctive norms such as believing people should engage in the behaviors and support for behavioral mandates were the strongest predictors of infection-prevention behavior. The results highlight how both data- and theory-driven approaches can increase understanding of complex human behavior.

Van Lissa et al., 2022, Patterns *3*, 100482
April 8, 2022 © 2022 The Author(s).
https://doi.org/10.1016/j.patter.2022.100482

CellPress

Patterns
CellPress
OPEN ACCESS

Article

Using machine learning to identify important predictors of COVID-19 infection prevention behaviors during the early phase of the pandemic

Caspar J. Van Lissa,[1,68,69,*] Wolfgang Stroebe,[2] Michelle R. vanDellen,[3] N. Pontus Leander,[2,67] Maximilian Agostini,[2] Tim Draws,[4] Andrii Grygoryshyn,[5] Ben Gützgow,[2] Jannis Kreienkamp,[2] Clara S. Vetter,[5] Georgios Abakoumkin,[6] Jamilah Hanum Abdul Khaiyom,[7] Vjolica Ahmedi,[8] Handan Akkas,[9] Carlos A. Almenara,[10] Mohsin Atta,[11]

(Author list continued on next page)

[1]Utrecht University, Utrecht, the Netherlands
[2]University of Groningen, Gronigen, the Netherlands
[3]University of Georgia, Athens, GA, USA
[4]Delft University of Technology, Delft, the Netherlands
[5]University of Amsterdam, Amsterdam, the Netherlands
[6]University of Thessaly, Volos, Greece
[7]International Islamic University Malaysia, Selangor, Malaysia
[8]University of Pristina, Pristina, Kosovo
[9]Ankara Science University, Ankara, Turkey
[10]Universidad Peruana de Ciencias Aplicadas, Lima, Peru
[11]University of Sargodha, Punjab, Pakistan
[12]Sabanci University, Tuzla, Turkey

(Affiliations continued on next page)

THE BIGGER PICTURE In the absence of a vaccine or cure, virus containment depended on individual-level compliance with behaviors recommended by the World Health Organization. We used machine learning to identify the most important indicators of compliance, based on a large international psychological survey and on country-level secondary data. The most important indicators were not the "usual suspects," such as personal threat of virus infection, but rather injunctive norms—namely, the belief that one's community should engage in such behavior and that society should take restrictive virus-containment measures. People who tend to engage in infection-prevention behaviors also tend to believe that general compliance is necessary to defeat the pandemic, which extends to endorsement of "ought" norms and support for behavioral mandates. These results highlight the potential to intervene by shaping social norms and expectations.

1 2 3 4 5 **Proof-of-Concept:** Data science output has been formulated, implemented, and tested for one domain/problem

SUMMARY

Before vaccines for coronavirus disease 2019 (COVID-19) became available, a set of infection-prevention behaviors constituted the primary means to mitigate the virus spread. Our study aimed to identify important predictors of this set of behaviors. Whereas social and health psychological theories suggest a limited set of predictors, machine-learning analyses can identify correlates from a larger pool of candidate predictors. We used random forests to rank 115 candidate correlates of infection-prevention behavior in 56,072 participants across 28 countries, administered in March to May 2020. The machine-learning model predicted 52% of the variance in infection-prevention behavior in a separate test sample—exceeding the performance of psychological models of health behavior. Results indicated the two most important predictors related to individual-level injunctive norms. Illustrating how data-driven methods can complement theory, some of the most important predictors were not derived from theories of health behavior—and some theoretically derived predictors were relatively unimportant.

This is an open access article under the CC BY license (http://creativecommons.org/licenses/by/4.0/).

Sabahat Cigdem Bagci,[12] Sima Basel,[13] Edona Berisha Kida,[8] Allan B.I. Bernardo,[14] Nicholas R. Buttrick,[15] Phatthanakit Chobthamkit,[16] Hoon-Seok Choi,[17] Mioara Cristea,[18] Sára Csaba,[19] Kaja Damnjanović,[20] Ivan Danyliuk,[21] Arobindu Dash,[22] Daniela Di Santo,[23] Karen M. Douglas,[24] Violeta Enea,[25] Daiane Gracieli Faller,[13] Gavan J. Fitzsimons,[26] Alexandra Gheorghiu,[25] Ángel Gómez,[27] Ali Hamaidia,[28] Qing Han,[29] Mai Helmy,[30,31] Joevarian Hudiyana,[32] Bertus F. Jeronimus,[2] Ding-Yu Jiang,[33] Veljko Jovanović,[34] Željka Kamenov,[35] Anna Kende,[19] Shian-Ling Keng,[36] Tra Thi Thanh Kieu,[37] Yasin Koc,[2] Kamila Kovyazina,[38] Inna Kozytska,[21] Joshua Krause,[2] Arie W. Kruglanksi,[39] Anton Kurapov,[40] Maja Kutlaca,[41] Nóra Anna Lantos,[19] Edward P. Lemay Jr.,[39] Cokorda Bagus Jaya Lesmana,[41] Winnifred R. Louis,[42] Adrian Lueders,[43] Najma Iqbal Malik,[11] Anton P. Martinez,[44] Kira O. McCabe,[45] Jasmina Mehulić,[35] Mirra Noor Milla,[32] Idris Mohammed,[46] Erica Molinario,[47] Manuel Moyano,[48] Hayat Muhammad,[49] Silvana Mula,[23] Hamdi Muluk,[31] Solomiia Myroniuk,[2] Reza Najafi,[50] Claudia F. Nisa,[13] Boglárka Nyúl,[19] Paul A. O'Keefe,[51] Jose Javier Olivas Osuna,[27] Evgeny N. Osin,[52] Joonha Park,[53] Gennaro Pica,[54] Antonio Pierro,[23] Jonas H. Rees,[55]

(Author list continued on next page)

[13]New York University Abu Dhabi, Abu Dhabi, United Arab Emirates
[14]De La Salle University, Metro Manila, Philippines
[15]University of Virginia, Charlottesville, VA, USA
[16]Thammasat University, Bangkok, Thailand
[17]Sungkyunkwan University, Seoul, South Korea
[18]Heriot Watt University, Edinburgh, Scotland
[19]ELTE Eötvös Loránd University, Budapest, Hungary
[20]University of Belgrade, Beograd, Serbia
[21]Taras Shevchenko National University of Kyiv, Kyiv, Ukraine
[22]Leuphana University of Luneburg, Lüneburg, Germany
[23]University "La Sapienza", Rome, Italy
[24]University of Kent, Canterbury, UK
[25]Alexandru Ioan Cuza University, Iași, Romania
[26]Duke University, Durham, NC, USA
[27]Universidad Nacional de Educacion a Distancia, Madrid, Spain
[28]Setif 2 University, Setif, Algeria
[29]University of Bristol, Bristol, UK
[30]Sultan Qaboos University, Muscat, Oman
[31]Menoufia University, Shibin Al Kawm, Al Minufiyah, Egypt
[32]Universitas Indonesia, Jawa Barat, Indonesia
[33]National Chung-Cheng University, Chiayi, Taiwan
[34]University of Novi Sad, Novi Sad, Serbia
[35]University of Zagreb, Zagreb, Croatia
[36]Monash University, Melbourne, VIC, Australia
[37]HCMC University of Education, Ho Chi Minh City, Vietnam
[38]Republic of Kazakhstan
[39]University of Maryland, College Park, MD, USA
[40]Durham University, Durham, UK
[41]Udayana University, Kuta Selatan, Indonesia
[42]The University of Queensland, St. Lucia, QLD, Australia
[43]University of Limerick, Limerick, Ireland
[44]The University of Sheffield, Sheffield, UK
[45]Carleton University, Ottawa, ON, Canada
[46]Usmanu Danfodiyo University Sokoto, Sokoto, Nigeria
[47]Florida Gulf Coast University, Fort Myers, FL, USA
[48]University of Córdoba, Córdoba, Spain
[49]University of Peshawar, Peshawar, Pakistan
[50]University of Padova, Padua, Italy
[51]Yale-NUS College, Singapore, Singapore
[52]HSE University, Moscow, Russia
[53]NUCB Business School, Nagoya, Japan
[54]University of Camerino, Camerino, Italy
[55]Bielefeld University, Bielefeld, Germany
[56]University of Siena - Arezzo Campus, Siena, Italy
[57]University of Exeter, Exeter, UK
[58]M. Narikbayev KAZGUU University, Nur-Sultan, Kazakhstan
[59]New York University Shanghai, Shanghai, China
[60]King Saud University, Riyadh, Saudi Arabia

(Affiliations continued on next page)

CellPress
OPEN ACCESS

Anne Margit Reitsema,[2] Elena Resta,[23] Marika Rullo,[56] Michelle K. Ryan,[2,57] Adil Samekin,[58] Pekka Santtila,[59] Edyta M. Sasin,[13] Birga M. Schumpe,[5] Heyla A. Selim,[60] Michael Vicente Stanton,[61] Samiah Sultana,[2] Robbie M. Sutton,[24] Eleftheria Tseliou,[6] Akira Utsugi,[62] Jolien Anne van Breen,[63] Kees Van Veen,[2] Alexandra Vázquez,[27] Robin Wollast,[41] Victoria Wai-Lan Yeung,[64] Somayeh Zand,[47] Iris Lav Žeželj,[20] Bang Zheng,[65] Andreas Zick,[55] Claudia Zúñiga,[66] and Jocelyn J. Bélanger[13]

[61]California State University East Bay, Hayward, CA, USA
[62]Nagoya University, Nagoya, Japan
[63]Leiden University, Leiden, the Netherlands
[64]Lingnan University, Tuen Mun, Hong Kong
[65]Imperial College London, London, UK
[66]Universidad de Chile, Santiago, Chile
[67]Wayne State University, Detroit, MI, USA
[68]Open Science Community Utrecht, Utrecht, The Netherlands
[69]Lead contact
*Correspondence: c.j.vanlissa@uu.nl
https://doi.org/10.1016/j.patter.2022.100482

INTRODUCTION

Behavioral measures are crucial in limiting the spread of infectious diseases. This was especially the case in the early phase of the coronavirus disease 2019 (COVID-19) pandemic between March and May 2020, when no vaccines were available. In this first phase of the pandemic, three infection-prevention behaviors were recommended by most governments: frequent handwashing, social distancing, and self-quarantining.[1] The efficacy of these measures for curbing the virus depends on the extent to which individuals engage in these behaviors. The COVID-19 pandemic represented a public health emergency with rich social- and system-level data available to evaluate engagement in compliance and focus research and future policy interventions on the most important predictors of such behaviors. Although, one approach might be to test whether a specific variable explains important variance in predicting health behaviors. The present work applies machine learning to a large psychological dataset, which was assembled in the early phase of the pandemic and enriched with country-level societal data in order to consider a wider pool of candidate variables. Our primary aim was to identify the most important predictors of infection-prevention behavior, given the available data; a secondary aim was to illustrate how inductive methods can help to inform crisis response.

Social and health psychology entered the pandemic with a large toolbox of personal-, social-, and societal-level theories that may all independently predict individual-level infection-prevention behavior to some extent. These individual health theories each involve some overlapping and some distinct predictors. However, when numerous disconnected studies use disparate research methods, levels of analysis, limited samples, and narrow contexts, it is difficult to compare the relative predictive utility of variables indicated by these theories. In other words, when any given study focuses only on the variables that fall within the scope of its theory, it is hard to tell how important the variables are relative to other variables considered by other theories (or variables not considered at all). Machine learning is a more holistic methodology as it can assess and compare a large number of potential predictors simultaneously, including theoretically relevant ones, and identify which predictors ultimately explain the most variance in the outcome measure of interest.

The aim of this study is to use machine learning to identify the most important predictors of infection-prevention behaviors during the early stages of the COVID-19 pandemic from a multinational, rapid-response survey. We combine multinational survey data, country-level secondary database integration, and machine-learning methods with the practical aim of identifying the most important predictors that could serve as targets for future research and behavioral interventions by governments and organizations such as the World Health Organization (WHO). This method offers a holistic evaluation of numerous candidate predictor variables. The candidate variables cover different theoretical domains so the results might speak to the relative importance of different theories as well as specific predictors. Moreover, the results of this inductive, exploratory approach might suggest promising avenues for future confirmatory research, to investigate the direction of causality, and could support the allocation of scientific resources toward the most promising predictors of compliance in future crises that resemble the current pandemic. Results can also provide input for theory development or refinement.[2]

Our study was conducted between March and May of 2020—that is, in the initial phase of the pandemic, several months before the first COVID-19 vaccine (Pfizer-BioNTech COVID-19) was approved by the US Food and Drug Administration in August of the same year. At the time, there was hope a future vaccine could bring an end to the pandemic, implying that behavioral measures were mainly an interim or short-term solution. However, by 2021, hopes surrounding vaccines had still not fully materialized, partly because the available vaccines waned in efficacy over time and across new virus strains, and because much of the global population remained unvaccinated (e.g., COVID-19 vaccine hesitancy has since become a major area of research).[3,4] By winter 2021, with new virus strains, recurring lockdowns, and the return of behavioral restrictions, the infection prevention behaviors recommended during the initial period of our study remained highly relevant.

Machine learning can identify candidate predictors

Machine learning can complement theory-driven approaches by identifying important determinants, or correlates, of a particular outcome, identifying blind spots in existing knowledge, and ranking predictors by their relative importance.[2] Machine

learning instead estimates predictive performance in new datasets and, thus, generalizability of the results. Further, it includes checks and balances to prevent spurious findings (i.e., overfitting; see Hastie et al.[5]). The random-forests algorithm, in particular, is free from certain assumptions of regression/correlation analysis, namely the assumption of linearity, absence of interactions, and normality of residuals. Random forests intrinsically capture non-linear associations and higher-order interaction effects and can account for multilevel data: the clustering variable can be included as a predictor, which allows for relationships to differ across clusters (e.g., if measurements or associations differ between countries).[6]

Our approach incorporated both individual-level (psychological) predictors and country-level (societal) variables. To identify key individual-level predictors of infection-prevention behaviors—at least during the initial phase of the pandemic—we launched a large-scale psychological survey in 28+ countries in the immediate weeks after the WHO declared COVID-19 a pandemic. The survey was designed with country-level database integration and machine learning in mind, and a separate team set out to perform machine-learning analysis in isolation of any confirmatory analysis. The *a priori* objective was to recruit tens of thousands of survey responses globally, to assess their attitudes toward and to society's prescriptions, and to examine how these factors relate to individual infection-prevention behaviors. The survey provided individual-level variables, such as basic demographic characteristics (e.g., gender, age, education, religiousness), brief self-report measures of various psychological factors (e.g., subjective states and well-being, work and financial concerns, societal attitudes, COVID-relevant attitudes and beliefs), and individual infection-prevention behaviors (e.g., handwashing, avoiding crowds).

Deductive and inductive approaches

Deductive research, or hypothesis testing, is the predominant focus of contemporary behavioral research. It tends to focus on a relatively narrow set of theoretically derived variables, and the results revolve around statistical inference: whether the theoretical hypotheses are supported by significant or reliable effects. In deductive research, less emphasis is placed on comprehensiveness or breadth of candidate predictors. Relatedly, the relative importance of different predictors is often of secondary importance, as is the model's predictive performance. Thus, although an advantage of deductive approaches is that they can be used to draw inferences about theoretical hypotheses, they also have specific limitations. These are particularly poignant in the context of the COVID-19 pandemic. To allocate scientific resources effectively in a crisis, it is important to cast a wide net among potential predictors and across different theories and to even include under-theorized factors to unearth potential blind spots in the extant literature. Inductive research—that is, rigorous exploratory work that identifies reliable patterns in data—is more suited to these demands.

In recent years, inductive research has been gaining traction as a technique to complement existing theories by identifying important omissions.[2] In particular, machine learning offers powerful new tools for systematic exploration that can identify relevant predictors and complex relationships that have eluded theoreticians.[7] Machine learning is an approach to data analysis that focuses on maximizing predictive performance. This involves the use of flexible models to find reliable patterns in data. Machine-learning models can distill a large set of candidate variables down to the ones that are most important in predicting the outcome of interest and also indicate the direction and shape of the marginal association between those predictors and the outcome. In a context where predictor variables are likely to be related to each other, machine learning is better suited to manage these complex relationships than, e.g., multiple regressions. Moreover, it incorporates checks and balances to prevent spurious findings.[5] However, it is important to note that inductive and deductive approaches are interwoven, as the set of variables used as input for a machine-learning analysis is typically based on theoretical considerations. Thus, as we describe below, we included in our survey a large set of candidate individual- and societal-level indicators of infection-prevention behavior that were of theoretical interest to our international group of psychology experts.

Relevant theory

Infection control that relies on individual compliance with health recommendations constitutes a public good. The main characteristic of public goods (e.g., clean air) is that people can benefit from it even if they have not contributed to its production or purchase. This creates the temptation to free ride on the contributions of others.[8,9] The COVID-19 pandemic has some characteristics of a public goods dilemma in that control of the virus can only be achieved if most members of society contribute to the effort.[8,9] However, a pandemic also differs from many other public goods dilemmas due to the immediate personal health threat of the virus: engaging in infection-prevention behavior not only reduces the societal spread of the infection, it also lowers individual infection risk. Accordingly, individual-level psychological factors could predict infection-prevention behavior even when individuals feel unobserved.[10–12] Thus, we might expect self-reported individual differences to predict compliance, such as perceived personal infection risk and vulnerability.

Beyond its potential as a public goods dilemma, the COVID-19 pandemic is also a health emergency with profound social, economic, and societal ramifications. In practical terms, millions of people were expected to lose their jobs, experience economic hardship, and suffer psychological strains as a result of lockdowns or self-quarantining.[13] More generally, an international group of behavioral scientists proposed various other psychosocial factors that may predict responses to the COVID-19 pandemic,[14] ranging from individuals' internal states to their societal attitudes and beliefs. This necessitated research that comprehensively (re-)examined potential predictors of infection-prevention behavior, with attention to the broad social, economic, and personal ramifications of the pandemic.

Our survey also included factors directly relevant to the domain of health behavior, such as those suggested by the Health Belief Model.[15,16] According to the Health Belief Model, two conditions must be met to motivate people to engage in COVID-19 infection-prevention behavior: they have to believe that they are at risk of contracting the virus and that engaging in the recommended virus-protection behaviors would be effective in reducing that risk.[15] A further assumption of this model is that the effect of perceived effectiveness of a health behavior will

be moderated by the perceived costs of engaging in that behavior. If the behavior is too effortful, people might not adopt it, even if they think that doing so would be effective. A second relevant theory is the Theory of Planned Behavior (TPB[17–19]). This more general psychological theory of behavior prediction posits that intentions to engage in a specific behavior would be predicted by three constructs: attitude toward the behavior (advantages and disadvantages), subjective norms (e.g., what is expected of me by important others), and perceived behavioral control (i.e., will I be able to do it).

Despite the potential relevance of health-behavior theories, they illustrate the aforementioned tendency of deductive research to focus on a narrow set of theoretical constructs. Other potentially important predictors, not germane to the given theory, might be overlooked. In line with this narrow focus, models based on such theories typically explain limited variance in the outcome variable. For example, a meta-analysis based on 185 independent tests of the TPB found that attitudes, subjective norms, and perceived control explain 39% of the variance in intention, with intention accounting for 22% of variance in behavior.[18] Although this descriptive performance is perceived as relatively strong in the field of social science, it still leaves room for potential predictors from other research domains. Thus, rather than focus exclusively on variables that target the health behavior, the present analysis casts a wide net by including psychological and societal factors that specifically pertain to the COVID-19 domain, as well as other factors whose relevance may generalize across domains.

The present study

We sought to distinguish important individual- and societal-level indicators of infection-prevention behavior using random forests.[6] The analysis is based on data from a large-scale psychological survey enriched with publicly available country-level secondary data (see Table 2 for an overview of the databases used). Random forests were used for their relatively competitive performance, computational inexpensiveness, and ease of interpretation.[20] The expected results consist of an estimate of predictive performance, which indicates how well the final model predicts infection-prevention behavior in a new sample, a ranking of predictors based on variable importance, which reflects their relative contribution to the model's predictive performance, and partial dependence plots, which reveal the direction and shape of each predictor's marginal association with the outcome.

The specific approach used in this paper maximized the reliability and generalizability of results in three ways. First, the data were split into a training sample, used to build the model, and a testing sample. The testing (or "hold-out") sample is never used in the initial analysis but rather is used to estimate the generalizability of the final model after analyses on the training sample are complete (*a priori* splitting of the dataset can be verified via the project's public historical record). This procedure helps to determine the model's predictive performance: in a classic deductive analysis, performance is traditionally expressed in terms of R^2, which reflects a theoretical model's descriptive performance, which is the percentage of variance in the outcome explained by the model in the data. In the machine-learning literature, by contrast, it is commonplace to estimate predictive performance by assessing R^2 in an independent test sample that was not used to estimate the model. Predictive performance reflects the generalizability of a model. Second, part of our global data collection efforts included the recruitment of paid subsamples from 20 countries that were representative of the population's age and gender distribution. Such sampling procedures can improve generalizability to the extent that it includes persons who might otherwise not participate as self-selected volunteers. Third, random forest is a specific machine-learning method that includes checks and balances to ensure reliability and generalizability of the results.[6] Random-forest analysis accomplishes this by splitting the training data into 1,000 bootstrap samples and estimating a regression-tree model on each of these bootstrap samples independently. Each regression tree in turn splits the sample recursively until the post-split groups reach a minimum size. A split is made by determining which predictor (out of a randomly selected subset of predictors) and value of that predictor maximizes the homogeneity of the post-split groups. Thus, a tree resembles a flowchart with relatively homogeneous end nodes. Interactions are represented by subsequent splits on different variables, non-linear effects are represented by repeated splits on the same variable, and random effects are represented by splits on the cluster variable (country) followed by splits on substantive variables. Naturally, each of these 1,000 models will include some spurious findings (overfitting). However, when the predictions from the 1,000 models are averaged, these spurious findings tend to balance out, thus leaving only the reliable patterns. Whether this approach is successful in identifying reliable and generalizable patterns can be objectively evaluated based on subsequent predictive performance on the hold-out (test) sample.

RESULTS

The Workflow for Open Reproducible Code in Science (WORCS) was used to make a reproducible archive of all analysis code and results, including fit tables and figures; see GitHub: https://github.com/cjvanlissa/COVID19_metadata.[21]

Data analytic plan

Prior to analysis, we split our data by randomly assigning 70% of observations to a training set and 30% of observations to a test set.[5] The test set was reserved exclusively for unbiased evaluation of the final model's predictive performance and was neither used nor examined during model building to prevent cross-contamination. Thus, all models were trained using the training set and evaluated using the test set. We applied a random-forest model using the ranger R package.[22] Random forests offer competitive predictive performance at a low computational cost, intrinsically capture non-linear effects and higher-order interactions, offer a single variable importance metric for multilevel categorical variables (such as country), and afford relatively straightforward interpretation of variable importance and marginal effects of the predictors.[6] With regard to the multilevel structure of the data, random forests inherently accommodate data nested within country, including cross-level interactions where a given predictor has a different effect in different countries.

The forest included 1,000 trees. The model had two tuning parameters: the number of candidate variables to consider at each

split of each tree in the forest and the minimum node size. The optimal values for these parameters were selected by minimizing the out-of-bag mean squared error (MSE) using model-based optimization with the R package tuneRanger.[23] The best model considered 31 candidate variables at each split and a minimum of six cases per terminal node.

The outcome metrics considered in the present study consist of (1) predictive performance, which reflects the model's ability to accurately predict new data, 2) variable importance, which reflects each predictor's relative role in accurately predicting the outcome measure, and 3) partial dependence plots, which indicate the direction and (non-)linearity of a specific marginal effect.[6] Predictive performance is, essentially, a measure of explained variance (R^2), except that in the machine-learning context, predictive performance is evaluated on the test sample, which was not used to estimate the model. Estimates of R^2 on the training sample should be interpreted as a measure of descriptive performance (i.e., how well the model describes the data at hand) and can be (severely) positively biased when used as an estimate of predictive performance in new data. Given that we recruited paid subsamples (age-gender representative) in 20 countries, we additionally computed predictive performance for the paid-only portion of the test sample to better examine the generalizability of our findings to the target population.

The relative importance of predictor variables is based on permutation importance: each predictor variable is randomly shuffled in turn, thus losing any meaningful association with the outcome, and the mean decrease in the model's predictive performance after permutation, as compared with the un-permutated model, is taken to reflect the (inverse) importance of that variable.[6]

The partial-dependence plots are generated using the metaforest R package.[4] Partial-dependence plots display the marginal (bivariate) association between each predictor and the outcome.[24] They are derived by computing predictions of the dependent variable across a range of values for each individual predictor while averaging across all other predictors using Monte Carlo integration.

Total variance explained

The random-forest model predicted a large proportion of the variance in self-reported infection-prevention behaviors in the full test sample (R^2_{test} = 0.523) as well as in the paid subsample (R^2_{rep} = 0.586). As these samples had not been used in model estimation, this indicates that the results are robust. Notably, the high predictive performance on the paid subsample indicates the generalizability of the findings. The explained variance in the training sample was of approximately the same magnitude (R^2_{train} = 0.518). This correspondence between training and testing R^2 indicates that the model successfully learned reliable patterns in the data and was not overfit.

The top 30 predictors, ranked by relative variable importance, are illustrated in Figure 1, along with an indication of whether the effect is generally positive, negative, or other (e.g., curvilinear). Table 1 serves as the legend for the variables illustrated in Figure 1. Table S3 provides full results of all 115 predictors, rank ordered by variable importance.

Consistent with expectations, the most important predictors of infection-prevention behavior included a mix of individual-level (survey) variables and country-level (database) indices. The shape of the bivariate marginal association between each predictor and the outcome is displayed in the partial-dependence plots (Figure 2). Recall that partial-dependence plots display the marginal relationship between one predictor and the outcome while averaging across all other predictors using Monte Carlo integration.[24] Note that the marginal predictions for the two levels of "leave for work" are identical; a denser Monte Carlo integration grid might show a small difference here but exceeds our computational resources.

Individual-level predictors

Social norms

By far the most important predictors of infection prevention behaviors were individual-level beliefs about how other people should behave and whether society should mandate infection-prevention behavior. The two strongest predictors were injunctive norms targeting infection prevention—namely, the belief that people in the community should engage in social distancing and self-isolation (ranked 1st) and their endorsement of extraordinary restrictive measures to contain the virus (e.g., mandatory quarantines and vaccination, reporting suspected infected individuals; ranked 2nd). The third strongest predictor was a prosocial willingness to protect vulnerable groups from the coronavirus (ranked 3rd). Respondents who complied with the norm to engage in infection-prevention behaviors indicated that they wanted to do their part to help other people cope with the pandemic. Other, related indicators included the descriptive normative belief that people in one's community do self-isolate and engage in social distancing (ranked 7th), a pro-social willingness to limit the economic consequences of the coronavirus on others (ranked 8th), and support for economic intervention (ranked 26th). Partial-dependence plots indicate that the injunctive ("should") norm had a positive, approximately exponential, marginal relationship with the outcome measure, whereas the other indicators had positive, approximately linear, marginal relationships.

Social and public behavior

The next most important indicators were behavioral correlates of the dependent measure, namely, self-reported days in the last week that the individual engaged in social and public contact. Each of these behaviors had a negative, approximately linear relationship with infection-prevention behaviors. This included the number of days that respondents reported leaving home (ranked 5th), the number of days in the past week they had in-person (face-to-face) contact with people who live outside their home, including "… immigrants" (ranked 4th), "… other people in general" (ranked 6th), and "… friends and relatives" (ranked 20th). Thus, higher in-person contact, which is inadvisable during a pandemic, generally corresponded with less infection-prevention behavior. In contrast, online (virtual) contact with friends and relatives—which does not violate social-distancing measures—positively predicted infection prevention behavior (ranked 25th).

Personal psychological factors

A third set of individual-level predictors thematically pertained to personal and psychological resources, and all had positive linear relationships with the outcome variable: a problem-focused coping style (ranked 9th), having high hopes that the COVID-19 situation would soon improve (ranked 11th), and a temporal focus

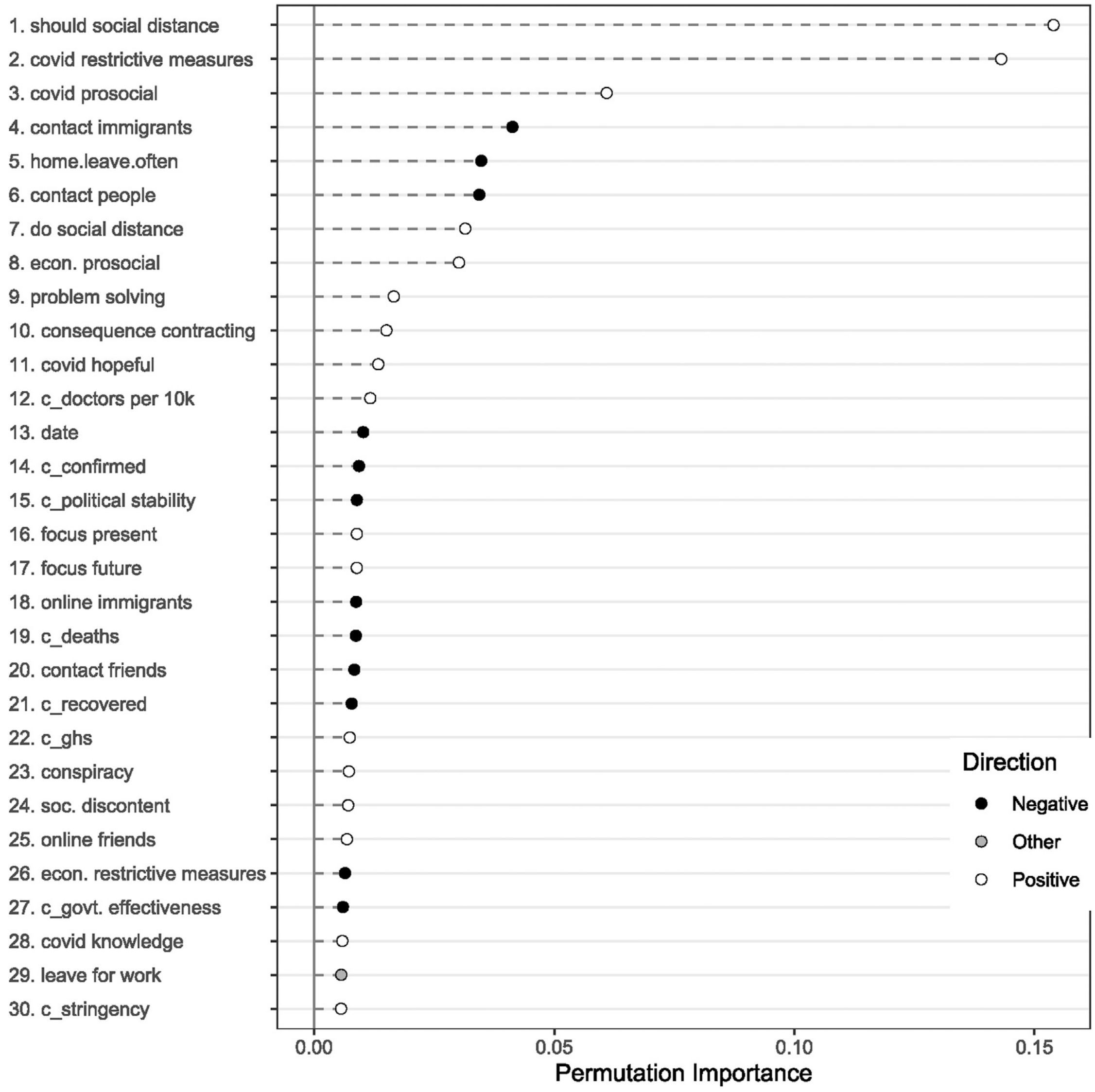

Figure 1. Machine-learning results for self-reported personal infection-prevention behavior
Variables ranked in order of relative importance.

on the present (ranked 16th) and/or the future (ranked 17th). Consistent with theories of health behavior,[25] the perceived personal consequences of COVID-19 infection ranked 10th. Relatedly, self-reported knowledge about COVID-19—important for risk-assessment—ranked 28th.

Several individual-level variables rounded out the bottom of the list. These are harder to interpret because of their lower variable importance and non-conclusive partial-dependence plots. Having to leave one's house for work (ranked 29th) had a slight negative association with infection-prevention behavior, perhaps because having to leave the house for extrinsic reasons hinders social distancing and self-isolation. The positive association between conspiracy beliefs and infection-prevention behavior (ranked 23rd) might seem paradoxical, as one might expect a negative association, if we had specifically measured belief in the conspiracy theory that the virus is a hoax. However, we instead assessed generic conspiracy beliefs[26]—whether respondents believe that politicians do not always disclose the motives behind their decisions, that important things happen without public knowledge, and that government agencies closely monitor citizens. It might be the case that participants who endorse these beliefs tend to take infection prevention into their own hands.

Country-level predictors

General societal conditions

Five (of 9) general societal indices were ranked among the important indicators of infection prevention behaviors. The most important country-level predictor was a WHO/OECD indicator of national health care resources and infrastructure: the number of doctors per 10,000 inhabitants (ranked 12th). Other country-level predictors were the Global Health Security index (ranked

Table 1. Brief descriptions of the top 30 predictors listed in Figure 1

	Variable	Brief description
1	should social distance	injunctive norm (right now, people in my area … "- … should self-isolate and engage in social distancing")
2	covid restrictive measures	support for severe collective virus-containment measures (3 items: mandatory quarantines, mandatory vaccinations, report people suspected to be infected with COVID-19)
3	covid pro-social	pro-social willingness to protect vulnerable groups from the coronavirus (4 items)
4	contact immigrants	days of in-person (face-to-face) contact with immigrants
5	home.leave.often	how many days in the last week did you leave your home?
6	contact people	days of in-person (face-to-face) contact with other people in general
7	do social distance	descriptive norm (right now, people in my area … "- … do self-isolate and engage in social distancing")
8	econ pro-social	pro-social willingness to protect vulnerable groups from economic consequences of the coronavirus (3 items)
9	problem solving	problem-focused coping style (3 items)
10	consequence contracting	how personally disturbing would it be if … "you were infected with coronavirus"
11	covid hopeful	"I have high hopes that the coronavirus situation will soon improve"
12	c_doctors_per10k	number of doctors per 10,000 residents (country-level; WHO)
13	date	date of survey participation (March 19–May 25).
14	c_confirmed	number of confirmed coronavirus infections (country-level; Johns Hopkins CSSE)
15	c_political stability	political stability and absence of violence/terrorism (country-level; WGI)
16	focus_present	temporal focus on the present moment
17	focus_future	temporal focus on the future
18	online_immigrants	days of online (virtual) contact with immigrants in the past week
19	c_deaths	number of confirmed COVID-19 deaths (country-level; Johns Hopkins CSSE)
20	contact friends	days of in-person (face-to-face) contact with friends and relatives in the past week
21	c_recovered	number of confirmed COVID-19 recoveries (country-level; Johns Hopkins CSSE)
22	c_ghs	global health security index: pandemic preparedness and health security (country-level; source: Global Health Security Index)
23	conspiracy	generic conspiracy beliefs (3 items)
24	societal discontent	concern about direction of society (3 items)
25	online friends	days of online (virtual) contact with friends and relatives in the past week
26	econ. restrictive measures	support for extraordinary governmental intervention in economy (3 items)
27	c_govt. effectiveness	government effectiveness (country-level; WGI)
28	covid knowledge	"How knowledgeable are you about the situation regarding the coronavirus?"
29	leave for work	"In the past week, did you leave your house for work?" (binary)
30	c_stringency	government COVID response tracker, measured across 17 policy indicators (country-level; source: OxCGRT)

Full details of each measure are provided in Table S3, as well as the survey codebook (OSF: https://osf.io/qhyue/?view_only=d60116c8090d4ec696bfaa9ea14b9432). Country-level variables are denoted with a c_ at the beginning of each variable name. Full variable descriptions are in the supplemental information.

22nd), which pertains to pandemic preparedness and general health security, and two (out of six) World Governance Indicators: political stability (15th) and government effectiveness (27th). Country-level COVID-19 policy stringency (i.e., severity of lockdown conditions) ranked 30th, which potentially illustrates the limits of government lockdowns in compelling individual-level behavior, relative to other predictors.

COVID-19 conditions

All three indicators of objective COVID-19 virus spread conditions in participants' countries at the time of participation were important indicators of infection-prevention behavior: the cumulative number of confirmed COVID-19 cases (ranked 14th), deaths (ranked 19th), and recoveries (ranked 21st). All three patterns were negative, indicating that self-reported infection-prevention behavior was lower among respondents who lived in countries with higher virus case counts, deaths, and recoveries on the day that they responded to the survey.

The effect of time

As our study covered a span of several weeks, time could be included as a predictor, operationalized as the calendar date of each survey response. The effect of time was negative (ranked 13th), indicating that self-reported infection-prevention behavior generally decreased between March and May 2020.

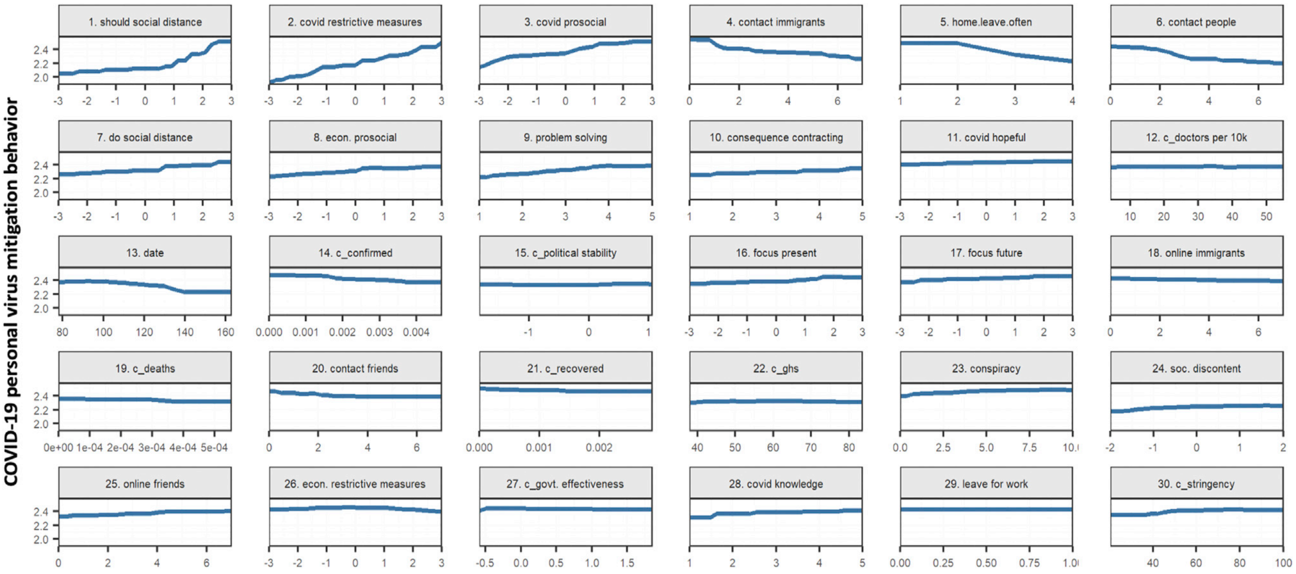

Figure 2. Partial-dependence plots depicting bivariate associations between each variable and infection-prevention behaviors

DISCUSSION

The present study used machine learning to identify and rank predictors of infection-prevention behavior among a wide set of potential candidates. After training on one sample, the resulting random-forest model predicted over 50% of the variance in self-reported infection-prevention behavior in a second (test) sample. This exceeds the standards for explained variance of social and health psychological theories, thus indicating that this data-driven approach can complement theoretical models. Moreover, whereas theoretical models typically focus on a limited, narrow set of relevant variables, the present machine-learning analysis identified additional, under-theorized predictors (e.g., temporal focus), thus offering complementary insights.

Who complies with infection-prevention behavior?

A coherent picture emerged from our analysis of the type of person that showed early compliance with the recommended set of infection=prevention behaviors. The underlying pattern of individual-level indicators could point to an intuitive understanding that infection control is a public good and to a conviction that the only way of virus mitigation involves widespread compliance with recommended behaviors. The compliant individuals appear to understand that factors such as personal risk (which was not indicated as highly important) is managed through similar efforts from others. If everybody engaged in infection-prevention behavior, the number of infected people in society would be reduced. Furthermore, if the people who did contract the virus maintained physical distancing, they would be less likely to infect others. This would explain why the strongest correlates of infection-prevention behavior were beliefs that others in the community should engage in social distancing and self-isolation and that society should take restrictive measures to enforce that behavior, such as mandatory quarantine, reporting people suspected to be infected, and (eventually) mandatory vaccination. Endorsement of such measures implies the prioritization of infection control over concerns about people's liberties and autonomy.

The descriptive normative belief, that other people in the community do engage in social distancing and self-isolation, also emerged as a relatively important predictor. It makes sense that individuals might be less motivated to comply if they were among a community of non-compliers. Furthermore, according to self-reports about their own behavior, compliant individuals did not engage in behavior that would be inconsistent with self-protection, such as leaving their homes or having personal contact with other people. If they had contact with their family and friends, it was not in face-to-face meetings, but online.

The findings also point to the idea that people who comply with recommended infection-prevention behaviors are forward-looking problem-solvers. That is, they tended to engage in a problem-focused coping style, focused on the present and the future (rather than dwell on the past), and maintained high hopes that the COVID-19 situation would soon improve. This optimistic view is important because these individuals were likely aware of the costs of these infection-prevention behaviors and perhaps needed psychological resources to alleviate these costs. In this vein, other important predictors were a pro-social willingness to self-sacrifice to protect vulnerable groups from the virus, to limit the economic consequences of COVID-19 on such groups, and to support collective interventions in the economy, such as tax increases. These results might also help understand the tension between members of society who do and do not engage in updated recommendations. Given that the largest predictor of infection-prevention behaviors—at least those originally recommended by the WHO—is the injunctive normative belief that one should participate in the behaviors, people who do not engage in those behaviors are likely to be seen as immoral or, at the very least, norm violators. In support of this, a large British survey indicated in September 2020—3 months after the WHO started to universally recommended mask wearing—that 58% of the mask wearers in Britain had severely negative attitudes toward

those who did not wear masks and that 68% of Brits who complied with lockdown rules had strong negative views about lockdown rule breakers. In fact, significant minorities who kept to the rules said that they "hated" those who did not.[27]

Aside from individual-level factors, several country-level indicators emerged as important predictors. This pattern of results is noteworthy for several reasons. First, because it means that there are meaningful between-country differences in compliance, which are partly explained by country-level characteristics. Second, the absence of the variable "country" from the top predictors indicates that there are no remaining between-country differences in compliance to be explained once the effect of the included country-level predictors is accounted for. Thus, it is unlikely that other between-country differences—such as collectivism/individualism—have a meaningful effect over and above a country's healthcare resources (e.g., number of doctors) and pandemic severity. Third, whereas it could be argued that the effect of individual-level predictors might be inflated due to common method bias, this explanation can be ruled out for the country-level predictors. The fact that these factors were among the most important predictors thus speaks to the robustness of the findings.

The findings regarding country-level predictors further suggest that infection control is a societal-level challenge, in that individual-level compliance with infection-prevention recommendations is more likely in a society that has the political stability and healthcare infrastructure to take effective action to contain the virus and treat people who have become infected. The findings regarding country-level indicators are consistent with this analysis: government stability and effectiveness, pandemic preparedness, healthcare resources (i.e., number of doctors), and lockdown stringency were all relatively important indicators of infection-prevention behavior.

Respondents in countries with higher confirmed COVID-19 infections, deaths, and recoveries reported less infection-prevention behavior themselves. Such findings might suggest reverse causality, as a country is likely to experience increased pandemic severity if its citizens do not endorse infection-prevention behaviors. Alternatively, it is possible that higher virus counts demotivate infection-prevention efforts—though, this assumes widespread individual-level knowledge about virus rates. Given that self-reported knowledge about COVID-19 was an important positive indicator, it is more plausible that in a society in which there is less compliance, there will be more infections, deaths, and recoveries.

Finally, one worrisome association is that time since the start of the pandemic, operationalized as date of participation, emerged as an important negative predictor of personal health behavior. This suggests that even in the early phase of the pandemic, there was already a decrease in compliance with governmental advice. It could be that with time, self-isolation and social distancing became unbearable for many people. This is consistent with the notion of "COVID-fatigue" and highlights the need to investigate what factors might promote more sustained adherence to infection-prevention behaviors.

Unexpected absences from top indicators

It is interesting to consider some of the other 85 variables that were not among the top indicators. From a health psychological perspective, it is surprising that the perceived personal likelihood of getting infected was not among the important predictors. Though, the perceived personal consequence of infection was ranked 10th. According to the Health Belief Model,[15] perceived vulnerability and severity are both central to health-threat appraisal. The fact that the perceived severity of getting infected was a highly ranked predictor, but perceived infection risk was not, might suggest that people's behavior is more strongly driven by expected consequences than probability. Alternatively, the link between compliance and infection risk might be smaller because people implicitly recognize that this risk is largely outside of their control to the extent that the pandemic constitutes a public goods dilemma.

Several other theoretically relevant variables that were absent from the most important predictors included loneliness and boredom, emotional and affective states experienced during the last week, subjective well-being, various forms of psychological and financial strain, and job insecurity. It is important to note, however, that the present analysis does not rule out the importance of these personal factors for other outcomes nor does it serve as evidence for a null effect.

No demographic variables emerged as especially important even though several are associated with increased risk of complications from COVID-19. For instance, elderly people are at higher risk to die from a COVID-19 infection and are therefore strongly advised to take great care.[28] Furthermore, there is reason to assume that social distancing and self-isolation present more of a dilemma to young rather than elderly people, especially those on a pension. For young people, the costs of social distancing and self-isolation are typically higher and—because they usually recover more easily from a COVID-19 infection—the rewards of those infection-prevention behaviors are smaller. Consistent with this argument, the media have framed the pandemic as a potential "intergenerational conflict of interest," where the young bear the brunt of the cost of containment measures while the elderly enjoy most of its benefits. It is therefore noteworthy that our analysis did not identify age as an important predictor. This finding is consistent with pre-registered research that similarly found no support for the intergenerational conflict of interest hypothesis.[29]

Limitations, strengths, and future directions

An important strength of this study is that the questionnaire used was designed by an interdisciplinary consortium of scientists from different countries. This resulted in a questionnaire with a broader scope than those guided by a singular theoretical perspective. It makes the resulting data ideally suited for a machine-learning analysis that can distill the most important predictors from many potential candidates. However, despite this broad scope, it is important to acknowledge that this study covered only a small fraction of available psychological and societal factors. Similar studies are recommended to identify other important predictors of virus prevention behaviors including related behaviors that emerged later in the pandemic, such as the wearing of face coverings and vaccination.

Another strength is the very large international sample, which made it possible to apply machine-learning methods to identify important patterns in the data. Additionally, the availability of an age-gender representative subsample improved the

generalizability of the findings. Finally, a noteworthy strength is that the variance explained by the model was consistently high, and approximately the same, in the sample used to train the model ($R^2_{train} = 0.52$), in the testing sample used to estimate the robustness of the findings ($R^2_{test} = 0.52$), and in the age- and gender-representative testing sample used to estimate generalizability of the findings to the target population ($R^2_{rep} = 0.59$). This indicates that the model captured reliable patterns in the data, without overfitting noise and spurious effects, and that it has high generalizability.

There are also limitations in the methods and sampling. A methodological trade off was made due to the urgency of the crisis: In order to respond rapidly to the pandemic onset in March 2020, with a large-scale cross-national study, while relying on volunteer efforts and limited funding, the choice was made to exclusively use self-report measures, which are easily translated and administered to large-scale samples at low cost. Of course, the use of self-report measures risks introducing variance due to the subjective nature of self-reports and common method bias between self-reported predictors and the outcome. A second methodological limitation—one shared with all non-experimental research—is the question of causality. For some of the included predictors, causal mechanisms may be known or suggested by theory. For others, future research will be needed to examine whether causal relations exist, and for others still, causality might be unlikely. We have taken care to discuss the associations observed through the lens of past theory. Since causality cannot be inferred from these results, the primary contribution of this study is the rapid reduction of a large number of candidate predictors to a smaller subset of those most strongly associated with the outcome of interest. This allows researchers to prioritize the most likely candidate predictors for future research and helps policy makers focus their efforts on the most influential predictors for which causal mechanisms are known or suspected. Conversely, it is also useful to know which factors are not strongly associated with virus-prevention behaviors, as policies that target these factors are unlikely to be effective. For some variables, causality might be unlikely, but these might still be helpful from a descriptive point of view, to decide who to target in interventions, or to contextualize the relative importance of other variables.

A third limitation pertains to the sampling: although efforts were made to recruit age-gender representative subsamples, even these subsamples will not be strictly representative of the target population. Moreover, they could be otherwise biased by other, potentially unknown characteristics—including the different virus strains and shifting societal responses of the pandemic. Nonetheless, the approximately stable model performance across all samples reduces the likelihood that generalizability to the target population would be substantially different.

The analysis of this study uses deductive methods to maximize predictive performance, typically explain more variance than purely deductive approaches, and, in the case of random forests, intrinsically capture non-linear effects and higher-order interactions, including between-country differences in effects. However, the results are harder to interpret than the parameters (e.g., regression coefficients and p values). We should note that the variables included in the PsyCorona survey were guided by theory, and thus our approach combines inductive and deductive approaches. Thus, although our application of machine learning is useful for gaining preliminary insights, it also capitalizes on a rich history of theorizing about what drives engagement in health behavior. However, although our study includes potentially important variables and theoretical areas, it is neither exhaustive nor conclusive. Inductive analysis can complement theories or provide an impetus for the development of new hypotheses, but the output is not yet a comprehensive theory. Nevertheless, the present research contributes to the literature by offering a large-scale cross-national psychological survey, enriched by database integration and analyzed using machine learning.

Given that external enforcement of infection-prevention behaviors is difficult, recommendations are most likely effective if they are internalized by individuals and supported by societal-level factors. The picture that emerges from this analysis is that early compliance with infection-prevention-behavior recommendations is partly psychological and partly contextual. Our findings suggest a strong emphasis on norms—both injunctive and descriptive—and the societal conditions enabling these norms.

Although the data collected describe infection-prevention behaviors during the beginning of the pandemic, they may be useful for understanding later patterns of behavior (e.g., low vaccine rates) or future crises that involve a combination of personal and societal risks. Health-behavior theories tend to focus on the intrapersonal factors that predict behavior, possibly because these seem proximal to the health behaviors of interest. However, our data suggest that these proximal factors may predict less variance in behavior than broader considerations of communal behavior. Future models may benefit from considerations of perceptions of norms in conjunction with personal risk when they are applied to other health behaviors as well.

Conclusions

We began with an assumption that control of the pandemic is analogous to a public goods dilemma, in that COVID-19 is a social challenge that, in the absence of a vaccine at the time of the study, could only be addressed if enough individuals engaged in infection-prevention behavior. In accordance with this assumption, social beliefs and societal factors, rather than exclusively personal psychological states, emerged as the main predictors in our analysis.

EXPERIMENTAL PROCEDURES

Resource availability

Lead contact

The lead contact for this paper is Dr. Caspar van Lissa, who may be contacted at c.j.vanlissa@uu.nl.

Materials availability

The full survey is available in the supplemental information, as well as codebooks and translation procedures for all languages (Tables S1 and S2). All analysis code is available in an online repository (GitHub: https://github.com/cjvanlissa/COVID19_metadata), which also includes a full historical record since the start of the project. This can be used to verify that the analysis proceeded transparently and straightforwardly; the random seed used to select participants for the test sample was established before access to data was obtained, and testing data were never used for model training.

Data and code availability

Original data and code have been deposited to Zenodo: https://doi.org/10.5281/zenodo.5948816.

Table 2. Summary of country-level databases

Database	Description
1 Johns Hopkins University COVID-19 Data Repository Center for Systems Science and Engineering (CSSE)[a]	number of confirmed COVID-19 infections, deaths, and recoveries by date per country
2 Global Health Security (GHS) Index[b]	country-level ratings of pandemic preparedness and general health security
3 World Health Organization (WHO) and Organization for Economic Cooperation and Development (OECD)[c]	country-level healthcare resources and health infrastructure
4 World Bank: World-wide Governance Indicators (WGI)[d]	per-country data on aggregate ratings of voice and accountability, regulatory quality, political stability and absence of violence, rule of law, government effectiveness, and control of corruption
5 Oxford COVID-19 Government Response Tracker (OxCGRT)[e]	governmental responses and policies with respect to COVID-19 by date per country

[a]Available at https://github.com/CSSEGISandData/COVID-19.[62]
[b]Available at https://www.ghsindex.org/.
[c]Available at https://apps.who.int/gho/data/node.main.HWF and https://stats.oecd.org/index.aspx?queryid=30183.
[d]Available at http://info.worldbank.org/governance/wgi/.
[e]Available at https://www.bsg.ox.ac.uk/research/research-projects/coronavirus-government-response-tracker.

Data re-use disclosure statement

The PsyCorona data were made available for theory-testing studies by the researchers who helped to collect the data. Portions of the PsyCorona data have been previously reported in specific hypothesis tests.[29–33] This machine-learning analysis was planned *a priori* as part of the onset of PsyCorona, is the only paper that uses inductive analysis, and is based on the total dataset.

PsyCorona survey: Recruitment and item selection

The survey was translated from English into 29 other languages by bilingual members of the international research team. It was distributed online during the early phase of the pandemic (March to May 2020), with most participants completing the survey in March and April (see Figure S1 for daily frequencies). Parallel sampling strategies were employed: convenience, snowball, and paid samplings. Given that age and gender were identified early as population vulnerability characteristics to the virus,[28,34] the self-selected samples were supplemented with paid subsamples that were representative of a given country's population distribution of age and gender. The panel firms Qualtrics Panels and WJX achieved age-gender representative samples in 20 countries (n ~ 1,000 per country): Argentina, Australia, Brazil, Canada, China, France, Germany, Italy, Japan, the Netherlands, Philippines, Romania, Russia, Serbia, South Africa, South Korea, Spain, Turkey, the United Kingdom, and the United States. Four additional countries only achieved gender representativeness due to insufficient access to the 55+ age group in Greece, Indonesia, Saudi Arabia, and Ukraine. These paid subsamples were used to improve the generalizability of the model.

In order to maximize project feasibility (e.g., each item was translated into 30 languages), increase survey breadth, and reduce participant burden, we used brief measures of each construct. Where possible, survey items were selected from established scales. Because the set of variables relevant to the pandemic (e.g., norms about handwashing, endorsement of stringent regulations for violating quarantine) did not exist prior to the pandemic, we crafted face-valid items to assess these constructs.

Although the PsyCorona study was designed and implemented prior to Van Bavel and colleagues'[14] discussion of candidate domains of inquiry for pandemic behavior, it touches on nearly all of these topics, including navigating threats, stress and coping, science communication, moral decision-making, and political leadership.

The survey covered three overarching themes. The first theme included personal factors that could affect individuals' capacity to respond to the virus, such as psychological coping and outlook, loneliness and deprivation, subjective emotional states, well-being, employment, and financial (in)security. The second theme pertained to social attitudes and norms, including general beliefs and attitudes about society, economic considerations, migrant attitudes and prejudice, perceived and preferred social norms for infection prevention, and endorsement of extraordinary virus containment and its economic rescue measures. The third theme pertained to virus-relevant personal concerns, values, and tendencies, including social contact and leaving the home, as well as the dependent variable of interest: self-reported engagement in voluntary infection-prevention behaviors recommended by the WHO. Personal factors adapted or informed by prior work included affective states (including valence and arousal[35]), boredom,[36] coping and avoidance,[37,38] financial strain,[39] loneliness,[40] neuroticism,[41] happiness and well-being,[42–44] time perception, management, and temporal focus,[45,46] working conditions, and job insecurity.[47–49] The social attitudes and norms domain included generic conspiracy beliefs and paranoia,[26,50] immigrant attitudes,[51–53] norm perceptions and preferences (adapted[54]), and societal discontent and disempowerment.[25,55] Virus-relevant personal concerns included perceived norms (both descriptive and injunctive, adapted[56]), virus-relevant beliefs and perceived knowledge, virus exposure risk and economic risk, and severity of virus and economic consequences (adapted[56,57]), trust in governmental pandemic communication and response (adapted[54,58,59]), and attitudes toward pro-social responses and extraordinary societal responses.[58] This list is not exhaustive; see Table S3 for a full list and item details and our OSF page for a full list of references for each item (OSF: https://mfr.de-1.osf.io/render?url=https://osf.io/7kfj5/?direct%26mode=render%26action=download%26mode=render).

Key demographic variables, such as age, gender, education level, and religiousness, were included as predictors. Country of residence was included as a categorical predictor. A summary table of all variables entered as predictors is available in (Table S3). Psychometric properties of scales, including reliability and the range of factor loadings, are available in Table S5. There was no evidence of multicollinearity among the continuous individual-level predictors, with all variance inflation factors between 1.11 and 2.66.

Infection-prevention behavior

Through May 2020, a set of three infection-prevention behaviors were advised across most countries and contexts: washing hands, avoiding crowds, and self-isolating/self-quarantining (wearing a face covering was not universally recommended by the WHO until June 2020[60]). Participants were presented with a single screen that read "to minimize my chances of suffering from coronavirus, I …" and indicated their agreement to "1. … wash my hands more often", "2 ….avoid crowded spaces," and "3 ….put myself in quarantine/self-isolate", each rated on a seven-point scale rated -3 (strongly disagree) to 3 (strongly agree). To ensure items could be combined into a unidimensional scale, we conducted Horn's parallel analysis.[61] Only one component had an Eigenvalue exceeding randomly permuted data. This component explained 70% of the variance in the three items, which is high. The three factor loadings were high and approximately equal in size (range: 0.78–0.89), indicating that it is justifiable to combine these three items into a mean score representing infection-prevention behaviors ($M = 2.20$, $SD = 1.00$, $\alpha = 0.75$). Note that the items were specifically framed to assess the behavioral intent to reduce the risk of infection, consistent with theories of health behavior that people engage

CellPress
OPEN ACCESS

in self-protective actions because they are perceived as instrumental for threat reduction.[56]

Data enrichment and data cleaning

We enriched the individual-level PsyCorona data with publicly available country-level datasets. These datasets were selected due to their international relevance for affording, shaping, or guiding individual-level behavioral responses to the virus: first, pandemic severity, as indicated by the number of cases, deaths, and recovered patients, second, pandemic-related policies including both pre-existing policies and ongoing governmental responses to the COVID-19 pandemic, and third, pandemic preparedness. Table 2 presents an overview of the databases. The time range in data collection afforded variability in the degree to which people in a given country were seeing cases and/or engaging in different containment policies. Where applicable, respondent's country-level data were matched to their date of participation (e.g., confirmed cases, lockdown severity). Altogether, there were 115 predictors (80 survey factors, 35 country-level factors).

We subsequently cleaned the data in several steps. First, to ensure that there was enough data on the country level, we excluded observations from countries that accounted for less than 1% of total observations. The final sample included N = 56,072 respondents across 28 countries (see Table S4 for samples that remained in the data). Second, we excluded any columns and rows from the data that had a proportion of missing values of more than 20%. Third, we computed mean scores for multiitem scales using the tidySEM R package.[62] For instance, responses to all 4 items on job insecurity[49] were summarized by creating a single composite score for job insecurity. Scales with low reliability were excluded (Cronbach's alpha < 0.65). See Table S5 for scale descriptive statistics, including reliability and range of factor loadings.

SUPPLEMENTAL INFORMATION

Supplemental information can be found online at https://doi.org/10.1016/j.patter.2022.100482.

ACKNOWLEDGMENTS

The lead author was funded by a NWO Veni Grant (NWO Grant Number VI.Veni.191G.090). This research received support from the New York University Abu Dhabi (VCDSF/75-71015), the University of Groningen (Sustainable Society & Ubbo Emmius Fund), and the Instituto de Salud Carlos III (COV20/00086) co-funded by the European Regional Development Fund (ERDF) ''A way to make Europe.''

AUTHOR CONTRIBUTIONS

The study was designed by C.J.V.L., W.S., M.R.v., N.P.L., M.A., J.B., B.G., and J.K. The manuscript was written by C.J.V.L., W.S., M.R.v., and N.P.L. Data analyses were performed by C.J.V.L., T.D., A.G., and C.S.V. G.A., J.H.A.K., V.A., H.A., C.A.A., M.A., S.C.B., S.B., E.B.K., A.B.I.B., N.B., P.C., H.-S.C., M.C., S.C., K.D., I.D., A.D., D.D.S., K.M.D., V.E., D.G.F., G.F.J., A. Gheorghiu, A. Gomez, J.G.-M., A.H., Q.H., M.H., J.H., B.F.J., D.-Y.J., V.J., Z.K., A. Kende, S.-L.K., T.T.T.K., Y.K., K.K., I.K., J.K., A.W.K., A. Kurapov, M.K., N.A.L., E.P.L. Jr., C.B.J.L., W.R.L., A.L., N.I.M., A.P.M., K.O.M., J.M., M.N.M., I.M., E.M., M.M., H.M., S.M., R.N., C.F.N., B.N., P.A.O., J.J.O.O., E.O., J.P., G.P., A.P., J.R., A.M.R., E.R., M.R., M.K.R., A.S., P.S., E.M.S., B.M.S., H.A.S., M.V.S., S.S., R.M.S., E.T., A.U., J.A.v.B., K.V.V., A.V., R.W., V.W.-I.Y., S.Z., I.L.Ž., B.Z., A.Z., and C.Z. contributed to project design, data collection, translation, and review of the manuscript.

DECLARATION OF INTERESTS

The authors declare no competing interests.

Received: December 7, 2021
Revised: December 23, 2021
Accepted: March 4, 2022
Published: March 9, 2022

REFERENCES

1. Omeife, H.O. (2020). Coronavirus: distancing and handwashing could lower flu rates, too. MedicalXpress https://medicalxpress.com/news/2020-04-coronavirus-distancing-handwashing-flu.html.
2. Van Lissa, C.J. (2018). Metaforest: Exploring Heterogeneity in Meta-Analysis Using Random Forests (0.1.2) [R-Package] (CRAN), https://cran.r-project.org/package=metaforest.
3. Aw, J., Seng, J.J.B., Seah, S.S.Y., and Low, L.L. (2021). COVID-19 vaccine hesitancy – a scoping review of the literature in high-income countries. Vaccines *9*, 900. https://doi.org/10.3390/vaccines9080900.
4. Wang, Q., Yang, L., Jin, H., and Lin, L. (2021). Vaccination against COVID-19: a systematic review and meta-analysis of acceptability and its predictors. Prev. Med. *150*, 106694.
5. Hastie, T., Tibshirani, R., and Friedman, J. (2009). The Elements of Statistical Learning: Data Mining, Inference, and Prediction (Springer).
6. Breiman, L. (2001). Random forests. Mach. Learn. *45*, 5–32.
7. Brandmaier, A.M., Prindle, J.J., McArdle, J.J., and Lindenberger, U. (2016). Theory-guided exploration with structural equation model forests. Psychol. Methods *21*, 566–582.
8. Stroebe, W., and Frey, B.S. (1982). Self-interest and collective action: the economics and psychology of public goods. Br. J. Soc. Psychol. *21*, 121–137.
9. Olson, M. (2009). The Logic of Collective Action: Public Goods and the Theory of Groups (Harvard University Press).
10. Oosterhoff, B., Palmer, C.A., Wilson, J., and Shook, N. (2020). Adolescents' motivations to engage in social distancing during the COVID-19 pandemic: associations with mental and social health. J. Adolesc. Heal. *67*, 179–185.
11. Deutsch, M., and Gerard, H.B. (1955). A study of normative and informational social influence upon individual judgments. J. Abnorm. Soc. Psychol. *51*, 629–636.
12. Hagger, M.S., Hardcastle, S.J., Chater, A., Mallett, C., Pal, S., and Chatzisarantis, N.L. (2014). Autonomous and controlled motivational regulations for multiple health-related behaviors: between-and within-participants analyses. Heal. Psychol. Behav. Med. *2*, 565–601.
13. Brooks, S.K., Webster, R.K., Smith, L.E., Woodland, L., Wessely, S., Greenberg, N., and Rubin, G.J. (2020). The psychological impact of quarantine and how to reduce it: rapid review of the evidence. Lancet *395*, 912–920.
14. Van Bavel, J.J., Baicker, K., Boggio, P.S., Capraro, V., Cichocka, A., Cikara, M., Crockett, M.J., Crum, A.J., Douglas, K.M., Druckman, J.N., et al. (2020). Using social and behavioural science to support COVID-19 pandemic response. Nat. Hum. Behav. *4*, 460–471.
15. Janz, N.K., and Becker, M.H. (1984). The health belief model: a decade later. Health Educ. Q. *11*, 1–47.
16. Abraham, C., and Sheeran, P. (2005). The health belief model. In Predicting Health Behaviour: Research and Practice with Social Cognition Models, *28–80*, M. Connor and P. Norman, eds. *(Open University Press)*.
17. Ajzen, I. (2005). Attitudes, Personality, and Behavior (Open University Press).
18. Armitage, C.J., and Conner, M. (2001). Efficacy of the theory of planned behaviour: a meta-analytic review. Br. J. Soc. Psychol. *40*, 471–499.
19. Robin, R., Mceachan, C., Conner, M., Taylor, N.J., and Lawton, R.J. (2011). Prospective prediction of health-related behaviours with the Theory of Planned Behaviour: a meta-analysis. Health Psychol. Rev. *5*, 97–144.
20. Strobl, C., Malley, K., and Tutz, G. (2009). An introduction to recursive Partitioning: rationale, application, and characteristics of classification and regression trees, bagging, and random forestst. Psychol. Methods *14*, 323–348.
21. Van Lissa, C.J., Brandmaier, A.M., Brinkman, L., Lamprecht, A.-L., Peikert, A., Struiksma, M.E., and Vreede, B.M.I. (2021). WORCS: A workflow for open reproducible code in science. Data Science *4*, 29–49. https://doi.org/10.3233/DS-210031.

22. Wright, M.N., and Ziegler, A. (2015). Ranger: A Fast Implementation of Random Forests for High Dimensional Data in C++ and R (CRAN), https://cran.r-project.org/package=metaforest.
23. Probst, P., Wright, M., and Boulesteix, A.-L. (2019). Hyperparameters and tuning strategies for random forest. Wiley Interdiscip. Rev. Data Min. Knowl. Discov. *9*, e1301. https://doi.org/10.1002/widm.1301.
24. Friedman, J.H. (2001). Greedy function approximation: a gradient boosting machine. Ann. Stat. *29*, 1189–1232.
25. Gootjes, F., Kuppens, T., Postmes, T., and Gordijn, E. (2021). Disentangling societal discontent and intergroup threat: explaining actions towards refugees and towards the state. Int. Rev. Soc. Psychol. *34*, 1–14.
26. Bruder, M., Haffke, P., Neave, N., Nouripanah, N., and Imhoff, R. (2013). Measuring individual differences in generic beliefs in conspiracy theories across cultures: conspiracy Mentality Questionnaire. Front. Psychol. *4*, 225.
27. (2020). Covid Lockdown Rules More Divisive than Brexit, Survey Finds. Guardian https://www.theguardian.com/world/2020/sep/11/covid-lockdown-rules-more-divisive-than-brexit-survey-finds.
28. (2020). COVID-19 guidance for older adults. Centers Dis. Control Prev. https://www.cdc.gov/aging/covid19-guidance.html.
29. Jin, S., Balliet, D., Romano, A., Spadaro, G., Van Lissa, C.J., Agostini, M., Bélanger, J.J., Gützkow, B., Kreienkamp, J., Abakoumkin, G., et al. (2021). Intergenerational conflicts of interest and prosocial behavior during the COVID-19 pandemic. Pers. Individ. Dif. *171*, 110535.
30. Han, Q., Zheng, B., Cristea, M., Agostini, M., Bélanger, J.J., Gützkow, B., Kreienkamp, J.; PsyCorona Collaboration, and Leander, N.P. (2021). Trust in government regarding COVID-19 and its associations with preventive health behaviour and prosocial behaviour during the pandemic: a cross-sectional and longitudinal study. Psychol. Med. 1–11. https://doi.org/10.1017/S0033291721001306.
31. Nisa, C.F., Bélanger, J.J., Faller, D.G., Buttrick, N.R., Mierau, J.O., Austin, M.M.K., Schumpe, B.M., Sasin, E.M., Agostini, M., Gützkow, B., et al. (2021). Lives versus livelihoods? Perceived economic risk has a stronger association with support for COVID-19 preventive measures than perceived health risk. Sci. Rep. *11*, 9669.
32. Romano, A., Spadaro, G., Balliet, D., Joireman, J., Van Lissa, C., Jin, S., Agostini, M., Bélanger, J.J., Gützkow, B., Kreienkamp, J., and Leander, N.P. (2021). Cooperation and trust across societies during the COVID-19 pandemic. J. Cross. Cult. Psychol. *52*, 622–642. https://doi.org/10.1177/0022022120988913.
33. Han, Q., Zheng, B., Agostini, M., Bélanger, J.J., Gützkow, B., Kreienkamp, J., Reitsema, A.M., van Breen, J.A.; PsyCorona Collaboration, and Leander, N.P. (2021). Associations of risk perception of COVID-19 with emotion and mental health during the pandemic. J. Affect. Disord. *284*, 247–255.
34. Wenham, C., Smith, J., Morgan, R., and Group, W. (2020). COVID-19: the gendered impacts of the outbreak. Lancet *395*, 846–848.
35. Russell, J.A. (1980). A circumplex model of affect. J. Pers. Soc. Psychol. *39*, 1161–1178.
36. Eastwood, J.D., Fahlman, S.A., Mercer-Lynn, K.B., and Flora, D.B. (2013). Development and validation of the multidimensional state boredom scale. Assessment *20*, 68–85.
37. Carver, C.S., Scheier, M.F., and Weintraub, J.K. (1989). Assessing coping strategies: a theoretically based approach. J. Pers. Soc. Psychol. *56*, 267–283.
38. Sexton, K.A., and Dugas, M.J. (2008). The cognitive avoidance questionnaire: validation of the English translation. J. Anxiety Disord. *22*, 355–370.
39. Selenko, E., and Batinic, B. (2011). Beyond debt, a moderator analysis of the relationship between perceived financial strain and mental health. Soc. Sci. Med. *73*, 1725–1732.
40. Hughes, M.E., Waite, L.J., Hawkley, L.C., and Cacioppo, J.T. (2004). A short scale for measuring loneliness in large surveys: results from two population-based studies. Res. Aging *26*, 655–672.
41. Hahn, E., Gottschling, J., and Spinath, F.M. (2012). Short measurements of personality – validity and reliability of the GSOEP big five inventory (BFI-S). J. Res. Pers. *46*, 355–359.
42. Abdel-Khalek, A. (2006). Measuring happiness with a single-item scale. Soc. Behav. Pers. *34*, 139–150.
43. Hershfield, H.E., Mogilner, C., and Barnea, U. (2016). People who choose time over money are happier. Soc. Psychol. Personal. Sci. *7*, 697–706.
44. Seligman, M. (2011). Flourish: A New Understanding of Happiness, Well-Being, and How to Achieve Them (Nicholas Brealey Publishing).
45. Macan, T.H. (1994). Time management: test of a process model. J. Appl. Psychol. *79*, 381–391.
46. Shipp, A.J., Edwards, J.R., and Lambert, L.S. (2009). Conceptualization and measurement of temporal focus: the subjective experience of the past, present, and future. Organ. Behav. Hum. Decis. Process. *110*, 1–22.
47. Konovsky, M.A., and Cropanzano, R. (1991). Perceived fairness of employee drug testing as a predictor of employee attitudes and job performance. J. Appl. Psychol. *76*, 698–707.
48. Porath, C., Spreitzer, G., Gibson, C., and Garnett, F.G. (2012). Thriving at work: toward its measurement, construct validation, and theoretical refinement. J. Organ. Behav. *33*, 250–275.
49. Van der Elst, T., De Witte, H., and De Cuyper, N. (2014). The Job Insecurity Scale: a psychometric evaluation across five European countries. Eur. J. Work Organ. Psychol. *23*, 364–380.
50. Schlier, B., Moritz, S., and Lincoln, T.M. (2016). Measuring fluctuations in paranoia: validity and psychometric properties of brief state versions of the Paranoia Checklist. Psychiatr. Res. *241*, 323–332.
51. (2019). User's Guide and Codebook for the ANES 2016 Time Series Study. Election Studies. https://electionstudies.org/wp-content/uploads/2018/12/anes_timeseries_2016_userguidecodebook.pdf.
52. ESS Round 7 Source Questionnaire (2014). American national election studies. https://www.europeansocialsurvey.org/docs/round7/fieldwork/source/ESS7_source_main_questionnaire.pdf.
53. Zavala-Rojas, D. (2014). Thermometer scale (feeling thermometer). In Encyclopedia of Quality of Life and Well-Being Research, A.C. Michalos, ed. (Springer), pp. 6633–6634. https://doi.org/10.1007/978-94-007-0753-5_1028.
54. Gelfand, M. (2019). Rule Makers, Rule Breakers: Tight and Loose Cultures and the Secret Signals that Direct Our Lives (Scribner).
55. Leander, N.P., Chartrand, T.L., and Wood, W. (2010). Mind your mannerisms: behavioral mimicry elicits stereotype conformity. J. Exp. Soc. Psychol. *47*, 195–201.
56. Stroebe, W. (2011). Social Psychology and Health (Open University Press).
57. Stroebe, W., Leander, N.P., and Kruglanski, A.W. (2017). Is it a dangerous world out there? The motivational bases of american gun ownership. Personal. Soc. Psychol. Bull. *43*, 1071–1085.
58. Van Zomeren, M., Postmes, T., and Spears, R. (2008). Toward an integrative social identity model of collective action: a quantitative research synthesis of three socio-psychological perspectives. Psychol. Bull. *134*, 504–535.
59. Stroebe, W., Kreienkamp, J., Leander, N.P., and Agostini, M. (2021). Do Canadian and U.S. American handgun owners differ? Canadian Journal of Behavioural Science *53* (3), 221–231. https://doi.org/10.1037/cbs0000243.
60. Advice on the use of masks in the context of COVID-19: Interim guidance. World Health Organization https://www.who.int/publications/i/item/advice-on-the-use-of-masks-in-the- community-during-home-care-and-in-healthcare-settings-in-the-context-of-the-novel-coronavirus-(2019-ncov)-outbreak (2020).
61. Horn, J.L. (1965). A rationale and test for the number of factors in factor analysis. Psychometrika *30*, 179–185.
62. Van Lissa, C.J. (2020). TidySEM: Generate Tidy SEM-Syntax (0.1.0.5) (CRAN), https://cran.r-project.org/package=metaforest.

Patterns, Volume *3*

Supplemental information

Using machine learning to identify important predictors of COVID-19 infection prevention behaviors during the early phase of the pandemic

Caspar J. Van Lissa, Wolfgang Stroebe, Michelle R. vanDellen, N. Pontus Leander, Maximilian Agostini, Tim Draws, Andrii Grygoryshyn, Ben Gützgow, Jannis Kreienkamp, Clara S. Vetter, Georgios Abakoumkin, Jamilah Hanum Abdul Khaiyom, Vjolica Ahmedi, Handan Akkas, Carlos A. Almenara, Mohsin Atta, Sabahat Cigdem Bagci, Sima Basel, Edona Berisha Kida, Allan B.I. Bernardo, Nicholas R. Buttrick, Phatthanakit Chobthamkit, Hoon-Seok Choi, Mioara Cristea, Sára Csaba, Kaja Damnjanović, Ivan Danyliuk, Arobindu Dash, Daniela Di Santo, Karen M. Douglas, Violeta Enea, Daiane Gracieli Faller, Gavan J. Fitzsimons, Alexandra Gheorghiu, Ángel Gómez, Ali Hamaidia, Qing Han, Mai Helmy, Joevarian Hudiyana, Bertus F. Jeronimus, Ding-Yu Jiang, Veljko Jovanović, Željka Kamenov, Anna Kende, Shian-Ling Keng, Tra Thi Thanh Kieu, Yasin Koc, Kamila Kovyazina, Inna Kozytska, Joshua Krause, Arie W. Kruglanksi, Anton Kurapov, Maja Kutlaca, Nóra Anna Lantos, Edward P. Lemay Jr., Cokorda Bagus Jaya Lesmana, Winnifred R. Louis, Adrian Lueders, Najma Iqbal Malik, Anton P. Martinez, Kira O. McCabe, Jasmina Mehulić, Mirra Noor Milla, Idris Mohammed, Erica Molinario, Manuel Moyano, Hayat Muhammad, Silvana Mula, Hamdi Muluk, Solomiia Myroniuk, Reza Najafi, Claudia F. Nisa, Boglárka Nyúl, Paul A. O'Keefe, Jose Javier Olivas Osuna, Evgeny N. Osin, Joonha Park, Gennaro Pica, Antonio Pierro, Jonas H. Rees, Anne Margit Reitsema, Elena Resta, Marika Rullo, Michelle K. Ryan, Adil Samekin, Pekka Santtila, Edyta M. Sasin, Birga M. Schumpe, Heyla A. Selim, Michael Vicente Stanton, Samiah Sultana, Robbie M. Sutton, Eleftheria Tseliou, Akira Utsugi, Jolien Anne van Breen, Kees Van Veen, Alexandra Vázquez, Robin Wollast, Victoria Wai-Lan Yeung, Somayeh Zand, Iris Lav Žeželj, Bang Zheng, Andreas Zick, Claudia Zúñiga, and Jocelyn J. Bélanger

Note. Some tables are too large to fit into a document and are thus linked separately. For the machine learning analysis, all analysis code and results are provided online at: DOI: 10.5281/zenodo.5948816. The PsyCorona survey details, including translation procedures and codebook in 30 languages, is available on the Open Science Framework at https://osf.io/qhyue/

Translation Procedures

Table S1. *PsyCorona Scale Translation Procedure*

Language	Translators	Backward Translation	Translation & Revision Team	Translation software	Other Translation Methods
Albanian	2	Yes	Yes	Yes	Backward translation used for some items only to check meaning.
Arabic	2	No	Yes	Yes	
Bengali	1	No	No	Yes	
Croatian	2	No	Yes		
Dutch	2	No	Yes	Yes	
English	*N/A*	*N/A*	*N/A*		*Survey and scales were developed in English*
Farsi	3	No	Yes	Yes	Backward translation used for some items only to check meaning, Initial survey with 3 translators, follow-up survey by 1 translator
French	2	No	Yes	Yes	
German	3	No	Yes	Yes	Searched for scales in papers and databases
Greek	2	No	Yes		
Hindi	1	No	No	Yes	
Hungarian	4	No	Yes		
Indonesian	2	No	Yes		Assisted using online dictionaries
Italian	2	No	Yes	Yes	
Japanese	2	No	Yes		
Korean	2	No	Yes		
Malay	2	No	Yes		Assisted using online dictionaries
Polish	4	Yes	Yes	Yes	
Portuguese	1	No	No	Yes	
Romanian	2	Yes	Yes		

Russian	2	No	Yes		
Serbian	2	No	Yes		
Simplified Chinese	2	No	Yes		
Spanish	4	Yes	Yes		
Thai	2	Yes	Yes		
Traditional Chinese	2	No	Yes		
Turkish	3	No	Yes		
Ukrainian	2	No	Yes	Yes	Backward translation used for some items only to check meaning.
Urdu	2	Yes			
Vietnamese	2	Yes	Yes	Yes	Assisted using online dictionaries

Note: Translators = Number of translators who worked on this scale. Backward Translation = One person translated the measure from English to the language, and a different person translated the scale from the language back to English to check for scale meaning. Translation & Revision Team = One person translated the scale from English to the language, and a second person revised this translation. Alternatively, each person translated the scale and worked together during the revision. Translation Software = Translators used a translation software in the process (e.g., Google Translate). Other Translation Methods = other methods used in the translation of the survey.

Table S2. *Potentially relevant translation issues*

Language	Translation Issues
Albanian	• We were careful to choose semantically correct translations over more literal ones aiming to accommodate cultural differences. In some cases it was needed to add more words for correct understanding.<br>• "Online vs. offline contact" was translated as "online vs. direct contact"<br>• The item about “belief in one God/more than one God” was translated as “belief in one God” as all the religions in Kosovo and Albania are monotheistic. While these kinds of beliefs were not separated for ex. in two items but were within one item, it may have been confusing for the subjects so the translation was adapted culturally.<br>• Items pertaining to political orientation (left/right wing) may not be relatable due to the terminology used. Longer descriptions may have been needed to explain the terms and ensure they are correctly understood.
Arabic	• Some words/phrases were changed or removed to accommodate regional religious beliefs.<br>• There wasn’t a word for ‘local community’ in Arabic so used the term ‘society’ instead.<br>• In the question where there is a distinction between should and do isolate/social distance myself, and want/have to … a formatting error caused the wrong term to be bolded in some items, but the wording was the same.
Bengali	*None*
Croatian	• Difficulty translating formidability items as the word formidability does not translate well. We adjusted the translation for better understanding.<br>• QID536 - “The events in my life are mainly determined by own actions” - we translated this as “The events in my life are mainly under my control” as this is more semantically correct.
Dutch	• Formidability was translated as 'powerful' as the Dutch word for formidability is rarely used.<br>• "Online vs. offline contact" was translated as "online vs. face-to-face contact"
English	*N/A*
Farsi	• Multiple questions did not translate well.<br>• Attitude about politics is a relatively western way to categorize people into groups.
French	*None*
German	• Some items were hard to translate. E.g. ‘community’ does not translate well.<br>• Semantically correct translations were sometimes chosen over more literal ones to accommodate cultural differences.
Greek	• “Online” in the item “In the past 7 days, how many days did you have **online** (video or voice) contact with …” was translated “Internet”.<br>• “Community” (in the present context) does not translate well into Greek.
Hindi	• Some items were too technical and did not translate well, so simpler translations conveying the meaning were chosen.
Hungarian	• Some items were difficult to translate accurately.
Indonesian	• Some items were difficult to translate accurately due to the inequality of meanings.

Italian	*None*	
Japanese	*None*	
Korean	*None*	
Malay	•	Some items were difficult to translate literally due to cultural considerations. E.g., the item about belief in one God/more than one God may be perceived as offensive to Malay Muslim when the item is being written as one item. Agreeing on the item may indicate that the individual believes in either one and this is unacceptable to the majority of Muslims in Malaysia.<br>• Items pertaining to political orientation (left/right wing) may not be relatable to many locally, due to the terminology used. Longer descriptions may be needed to explain the term to ensure the terms could be understood correctly.
Polish	•	Tightness-looseness construct was difficult to translate.
Portuguese	*None*	
Romanian	*None*	
Russian	•	Tightness-looseness construct is difficult to express in Russian.<br>• The terms "economic left-right" and "libertarian-authoritarian" make little sense without explanation to most Russians.
Serbian	•	Difficulty translating formidability items.<br>• Identification item translated as *I feel close to* instead of *I identify with*.
Simplified Chinese	*None*	
Spanish	•	Care taken when finding equivalence between standard Spanish and Latin American Spanish.
Thai	•	Difficulty in translating cross-cultural research terms.<br>• Some items were difficult to translate literally and accurately.<br>• Questions about bodies were confusing.
Traditional Chinese	•	Translated “in my country” to “in the place I live” in order to accommodate both Taiwan and Hong Kong (which is not a country, but a special administrative region).
Turkish	*None*	
Ukrainian	•	Questions about ‘bodies’ were confusing since the metaphor itself might not have been fully clear for the local population.<br>• The same concerns formidability. Many sentences had to be restructured in order to save the meaning of the question.
Urdu	*None*	
Vietnamese	•	Some translated items were difficult to express accurately in Vietnamese due to political and social issues (eg. protest/ protesting) and some were not popular to most Vietnamese people (eg. economic left-right or libertarian - authoritarian).

Table S4. Samples in the 28 countries that remained in the data after cleaning.

Country	n	Female	Male	Gender: other	Primary education	Secondary education	Vocational education	Some higher edu	Bachelor's degree	Master's degree	PhD
Ukraine	1433	0.603	0.396	0.001	0.004	0.091	0.134	0.386	0.108	0.22	0.057
Italy	2006	0.602	0.393	0.004	0.006	0.064	0.052	0.503	0.116	0.216	0.043
Greece	2875	0.675	0.323	0.002	0.006	0.017	0.048	0.25	0.379	0.232	0.068
Romania	2704	0.609	0.388	0.003	0.013	0.242	0.032	0.252	0.283	0.154	0.024
Indonesia	2410	0.509	0.486	0.005	0.009	0.351	0.06	0.048	0.37	0.128	0.034
Malaysia	895	0.712	0.286	0.002	0.002	0.056	0.009	0.12	0.534	0.229	0.049
Philippines	1530	0.564	0.425	0.011	0.01	0.077	0.065	0.108	0.555	0.126	0.058
Argentina	1412	0.565	0.431	0.004	0.01	0.233	0.142	0.28	0.241	0.053	0.041
Russia	1438	0.612	0.384	0.003	0.004	0.079	0.195	0.45	0.088	0.133	0.05
USA	11048	0.62	0.373	0.007	0.033	0.094	0.056	0.196	0.389	0.179	0.053
Canada	1538	0.574	0.416	0.01	0.02	0.174	0.109	0.205	0.31	0.141	0.042
Japan	1326	0.474	0.522	0.004	0.002	0.173	0.039	0.334	0.371	0.059	0.02
Egypt	1158	0.841	0.157	0.002	0.007	0.196	0.026	0.477	0.246	0.034	0.012
Netherlands	2409	0.623	0.371	0.007	0.018	0.122	0.183	0.222	0.133	0.234	0.088
Saudi Arabia	1468	0.527	0.463	0.01	0.015	0.192	0.061	0.101	0.493	0.099	0.039
France	1801	0.581	0.414	0.005	0.027	0.145	0.195	0.186	0.111	0.189	0.147
Spain	3203	0.627	0.368	0.006	0.014	0.12	0.158	0.299	0.253	0.106	0.05
Germany	1690	0.565	0.43	0.005	0.011	0.109	0.314	0.179	0.133	0.202	0.053
United Kingd	1935	0.612	0.383	0.005	0.008	0.193	0.132	0.191	0.258	0.158	0.06
South Korea	1452	0.57	0.427	0.003	0.005	0.03	0.015	0.403	0.421	0.097	0.03
Turkey	1826	0.604	0.395	0.002	0.008	0.015	0.208	0.107	0.465	0.153	0.045
Kazakhstan	812	0.562	0.437	0.001	0.001	0.041	0.041	0.302	0.268	0.268	0.079
Australia	1216	0.535	0.46	0.005	0.013	0.22	0.164	0.171	0.296	0.101	0.034
Kosovo	830	0.838	0.162	0	0.004	0.078	0.045	0.299	0.345	0.195	0.034
Brazil	1395	0.577	0.422	0.001	0.02	0.241	0.092	0.339	0.182	0.096	0.029
Poland	718	0.832	0.154	0.014	0.014	0.331	0.059	0.089	0.12	0.331	0.055
Republic of S	2122	0.661	0.337	0.002	0.013	0.17	0.268	0.121	0.248	0.141	0.039
South Africa	1422	0.568	0.429	0.004	0.017	0.189	0.071	0.361	0.283	0.061	0.018

Table S5: Scale descriptive statistics after combining PsyCorona survey items

Subscale	Items	n	mean	sd	min	max	skew	skew_2s	kurt	kurt_2se	Reliabili	Interpret	min_load	max_load
disc	3	55979	0.64	0.77	-2	2	-0.45	-21.51	0.15	3.55	0.68	Questionable	0.41	0.84
jbinsec	4	46018	-0.42	1.08	-2	2	0.38	16.71	-0.57	-12.39	0.81	Good	0.63	0.88
pfs	3	55962	0.12	1.05	-2	2	-0.1	-4.77	-0.66	-15.95	0.85	Good	0.65	0.92
fail	3	55981	-0.01	0.86	-2	2	-0.02	-0.92	-0.18	-4.37	0.66	Questionable	0.48	0.72
lone	3	56005	2.39	1.02	1	5	0.43	20.68	-0.51	-12.33	0.82	Good	0.76	0.82
probsolving	3	55976	3.7	0.85	1	5	-0.48	-23.35	0.15	3.63	0.84	Good	0.77	0.86
posrefocus	3	55973	3.1	0.91	1	5	-0.16	-7.88	-0.17	-4.1	0.85	Good	0.75	0.85
c19proso	4	55979	0.84	1.23	-3	3	-0.55	-26.37	0.16	3.84	0.77	Acceptable	0.56	0.8
c19perbeh	3	55982	2.19	1	-3	3	-1.88	-91.01	4.45	107.54	0.75	Acceptable	0.59	0.95
c19rca	3	55975	1.48	1.34	-3	3	-1.01	-48.75	0.78	18.81	0.71	Acceptable	0.59	0.81
ecoproso	4	55910	0.63	1.33	-3	3	-0.51	-24.82	0.05	1.13	0.86	Good	0.67	0.84
ecorca	3	55900	-0.1	1.41	-3	3	-0.15	-7.13	-0.43	-10.36	0.65	Questionable	0.59	0.68
bordeom	3	55970	1.37	1.29	-3	3	0	0.19	-0.35	-8.53	0.53	Poor	0.07	0.96
migrantthreat	5	55643	5.43	2.32	1	10	-0.18	-8.82	-0.68	-16.25	0.91	Excellent	0.75	0.89
cognitive test	3	55963	1.96	0.38	1	3	-0.78	-37.56	-0.3	-7.2	0.27	Unacceptable	0.22	0.47
neuro	3	55946	0.08	1.3	-3	3	-0.02	-1.21	-0.38	-9.11	0.69	Questionable	0.55	0.86
para	3	55901	3.93	2.19	0	10	0.29	14.02	-0.21	-4.96	0.69	Questionable	0.41	0.91
consp	3	55710	6.56	2.1	0	10	-0.46	-22.25	-0.03	-0.69	0.73	Acceptable	0.47	0.83

Fig. S1. Distribution of participation dates.

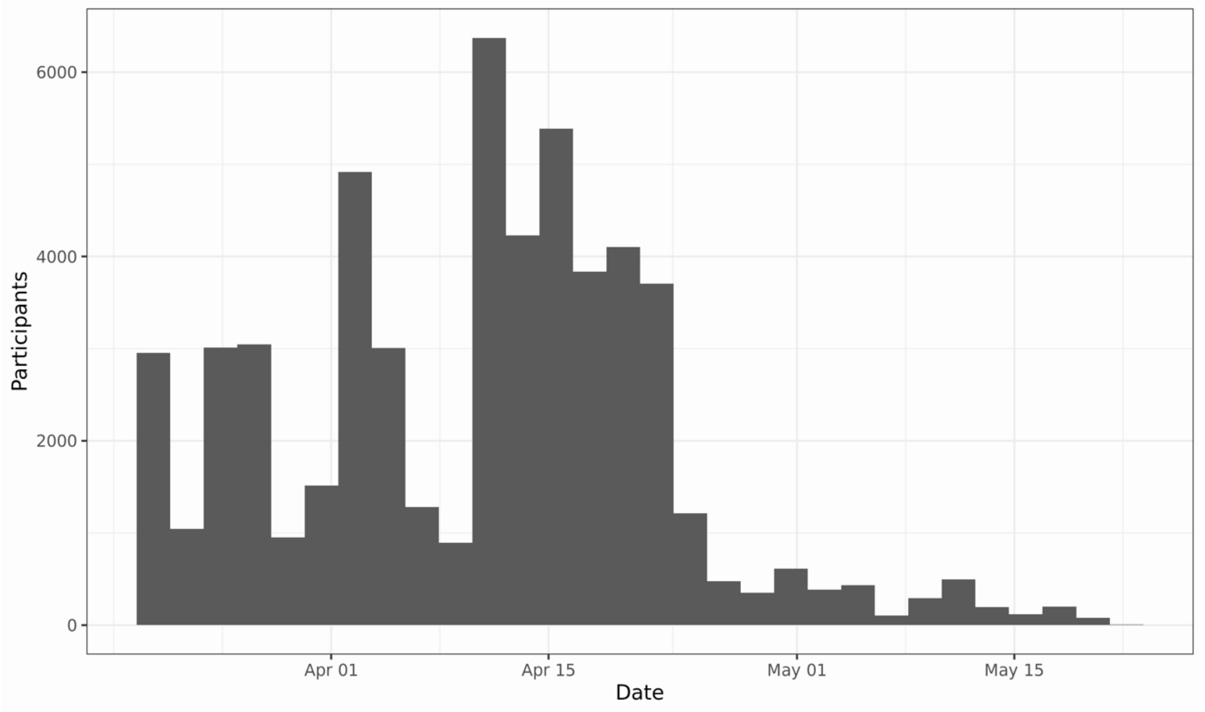

Supplement: Document S2. Article plus supplemental information [file mmc3.pdf]
